# Mesenchymal Stromal Cell-Conditioned Medium for Skin Diseases: A Systematic Review

**DOI:** 10.3389/fcell.2021.654210

**Published:** 2021-07-23

**Authors:** Trinidad Montero-Vilchez, Álvaro Sierra-Sánchez, Manuel Sanchez-Diaz, Maria Isabel Quiñones-Vico, Raquel Sanabria-de-la-Torre, Antonio Martinez-Lopez, Salvador Arias-Santiago

**Affiliations:** ^1^Department of Dermatology, Virgen de las Nieves University Hospital, Granada, Spain; ^2^Biosanitary Institute of Granada (ibs.GRANADA), Granada, Spain; ^3^Cell Production and Tissue Engineering Unit, Virgen de las Nieves University Hospital, Andalusian Network of Design and Translation of Advanced Therapies, Granada, Spain; ^4^Department of Dermatology, Faculty of Medicine, University of Granada, Granada, Spain

**Keywords:** advanced therapy, conditioned medium, dermatology, mesenchymal stem cells, stem cells

## Abstract

The skin is the largest organ of the human body, and its dysfunction is related to many diseases. There is a need to find new potential effective therapies for some skin conditions such as inflammatory diseases, wound healing, or hair restoration. Mesenchymal stromal cell (MSC)-conditioned medium (CM) provides a potential opportunity in the treatment of skin disease. Thus, the objective of this review is to evaluate the uses of MSC-CM for treating skin diseases in both animal and human models. A systematic review was conducted regarding the use of MSC-CM for treating skin conditions. One hundred one studies were analyzed. MSC-CM was evaluated in wound healing (55), hypertrophic scars (9), flap reperfusion (4), hair restoration (15), skin rejuvenation (15), and inflammatory skin diseases (3). MSC-CM was obtained from different MSC sources, mainly adipose tissue, bone marrow, and umbilical cord blood. MSC-CM was tested intravenously, intraperitoneally, subcutaneously, intradermally or intralesionally injected or topically applied. MSC-CM was used in both animals and humans. MSC-CM improved wound healing, hair restoration, skin rejuvenation, atopic dermatitis, and psoriasis in both animals and humans. MSC-CM also decreased hypertrophic scars and flap ischemia in animal models. In conclusion, MSC-CM is a promising therapy for skin conditions. Further studies are needed to corroborate safety and effectiveness and to standardize CM manufacturing.

## Introduction

Mesenchymal stromal cells (MSCs) are a type of multipotent adult stem cells that have the potential to proliferate, self-regenerate, and differentiate into multiple cell lineages (Dominici et al., 2006). They can be isolated from several sources such as bone marrow (BM-MSCs), adipose tissue (AT-MSCs), umbilical cord (UC-MSCs), amnion, placenta, or dental pulp (Bogatcheva and Coleman, 2019). MSCs are considered to be one of the most promising therapeutic options in cell therapy and tissue engineering, as they can be used for treating skin, cardiovascular, hematological, neurological, bone, and cartilage diseases (Wong et al., 2015; Kim et al., 2017; Hu et al., 2018; Martinez-Lopez et al., 2020).

It has been observed that the beneficial effects of MSCs are due not only to their multipotent ability but also to their secreted cytokines and growth factors (Caplan, 2017). Cell-free preparations have several advantages over cell therapy, as they can be obtained more easily and more economically and can be manufactured, packaged, and transported straightforwardly (Yang et al., 2021). Moreover, cell-free preparations do not have adverse events associated with cell administration such as rejection, tumorigenic, thrombogenic, ossification, or calcification risk (Bogatcheva and Coleman, 2019; Yang et al., 2021). The risk of malignant transformation of MSCs is a great concern, as MSCs therapy involves *ex vivo* production and expansion of cell lines, although the spontaneous malignant transformation of human MSCs has not been completely proved and there are many studies that have demonstrated that MSCs, even after physical and chemical stress, undergo senescence rather than become tumorigenic (Caplan et al., 2019). MSCs activate the host innate immune systems and the coagulation, increasing the expression of procoagulant tissue factor and demonstrating a procoagulant effect after MSC contact with blood in *in vitro* investigations. Infusion reactions and thromboembolism have been reported when using intravascular MSC products. A possible proposed solution to this problem is diluting or treating the MSCs with tissue factor pathway-blocking reagents. Moreover, hemocompatibility testing and optimal product delivery are important for designing safer MSC therapies. Other experimental intravascular therapies, such as islets, hepatocytes, and products derived from MSCs, could also improve MSC safety (Moll et al., 2019). The activation of the immune response could also lead to rejection. There are many investigations that focus on strategies to evade immune recognition, such as human leukocyte antigen (HLA)-matched cells or pharmaceutical immunosuppression (Moll et al., 2019, 2020). Cell-free preparation could help to reduce this risk of malignant transformation, thrombogenic risk, and rejection.

The molecules secreted by stromal cells are referred to the stromal cell secretome and include proteins, microRNA, growth factors, antioxidants, proteasomes, and exosomes (Maguire, 2013). The stromal cell culture media that comprise the secretome are known as the conditioned media (CM), and they are considered to be an abundant resource of paracrine factors (Pawitan, 2014; Mizukami and Yagihashi, 2016). The paracrine factors secreted *in vitro* include vascular endothelial growth factor (VEGF), hepatocyte growth factor (HGF), insulin-like growth factor-1 (IGF-1), IGF−2, and stromal cell-derived factor 1 (SDF−1) (Ratajczak et al., 2012; Deng et al., 2018). The administration of these factors to the site of an injured organ increases its metabolic activity and oxygen supply and remodels the extracellular matrix (Ratajczak et al., 2012).

The skin is the largest organ of the human body, and its dysfunction is linked to several diseases (Montero-Vilchez et al., 2021). MSCs provide a supply of new cells for epidermal homeostasis, hair cycling, and repairing injured tissue (Kim et al., 2017; Guo et al., 2020). MSCs-CM also provide a potential opportunity in the treatment of skin disease, and there is increased evidence justifying its use for the treatment of cutaneous conditions such as wound healing, hair growth, inflammatory skin diseases, or skin rejuvenation. Thus, the objective of this review is to evaluate the use of MSC-CM for treating skin diseases in both in animals and humans.

## Materials and Methods

### Search Strategy

A literature search was performed using Medline, Scopus, Embase, and ClinicalTrials.gov from conception to October 2020, following PRISMA Guidelines (Supplementary Material). The following search terms were used: [(MSC) OR (Mesenchymal Stem Cell) OR (Mesenchymal Stromal Cell) OR (Multipotent Stem Cell) OR (Multipotent Stromal Cell) OR (Stem Cell)] AND [(Conditioned Medium) OR (Conditioned Culture Media)] AND [(skin) OR (dermatology)].

### Inclusion and Exclusion Criteria

The search was limited to (i) human or animal data, (ii) *in vivo* studies, (iii) using MSC-CM for skin conditions, and (iv) articles written in English or Spanish.

All types of epidemiological studies (clinical trials, cohort studies, case–control studies, and cross-sectional studies) regarding MSC-CM use for skin conditions were included and analyzed. Reviews, guidelines, protocols, and conference abstracts were excluded.

### Study Selection

Two researchers (TMV and AML) independently reviewed the titles and abstracts of the articles obtained in the first search to assess relevant studies. The full texts of all articles meeting the inclusion criteria were reviewed, and their bibliographic references were checked for additional sources. The articles considered relevant by both researchers were included in the analysis. Disagreements about inclusion or exclusion of articles were discussed until a consensus was reached. If no consensus was reached, resolution was achieved by discussion with a third researcher (SAS).

### Variables

The variables assessed were MSC source, passage number and percentage of confluence, model tested, treatment group, route of administration, sample size, assessment, outcomes, and adverse events.

## Results

**FIGURE 1:**
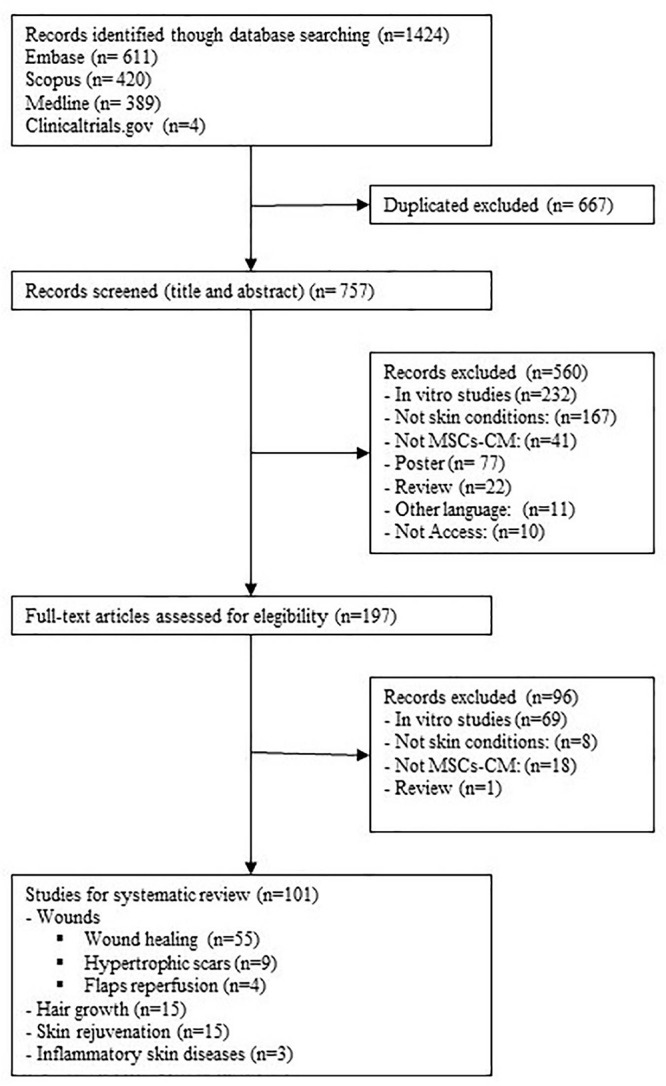
Flow diagram of the study selection process.

Our literature search identified 1,422 references, 757 after removing duplicates. After the title and abstract were screened, 197 records underwent full-text review. A total of 96 records were excluded because they did not investigate MSC-CM treatment *in vivo* skin conditions. Other reasons for exclusion along with the flowchart are shown in Figure 1. Ultimately, 101 studies met the eligible criteria.

Mesenchymal stromal cell-CM has been tested in several skin conditions: wound healing, hypertrophic scars, flap and graft reperfusion, hair restoration, aesthetic applications, and cutaneous inflammatory diseases.

### Wounds

#### Preclinical Studies

Fifty-three studies evaluated the effects of MSC-CM on wound healing, 58.49% (31/53) evaluated outcomes in non-diabetic wounds, and 35.85% (19/53) in diabetic wounds, including three studies that evaluated wound healing in both diabetic and non-diabetic animals (Fong et al., 2014; Tam et al., 2014; Raj et al., 2019). Moreover MSC-CM has also been tested on burn wounds (5.66%, 3/53), infected wounds (3.77%, 2/53), and radiation-induced wounds (1.89%, 1/53).

##### Non-diabetic wounds

Thirty-one studies evaluated the potential of MSC-CM for treating non-diabetic wounds (Table 1). To obtain CM, cells were mainly isolated from human tissues (74.19%, 23/31): from adipose tissue (Cho et al., 2010; Heo et al., 2011; Deng et al., 2017; Yuan et al., 2018; Chen et al., 2021) (21.74%, 5/23), amnion (Yoon et al., 2010; Jun et al., 2014; Park et al., 2018; He et al., 2020) (17.34%, 4/23), umbilical cord blood (Lee et al., 2011; Dong et al., 2017; Raj et al., 2019; Rong et al., 2019) (17.34%, 4/23), bone marrow (Tamari et al., 2013; Chen et al., 2014; Ahangar et al., 2020) (13.04%, 3/23), and Wharton’s jelly (Fong et al., 2014; Tam et al., 2014; Sabzevari et al., 2020) (13.04%, 3/23). Human dental pulp (Zhou et al., 2020) and skin (Robert et al., 2019) were also used. Moreover, murine, swine, caprine, canine, deer, and rabbit tissues were also employed (Chen et al., 2008; Templin et al., 2009; Sun et al., 2014; Mehanna et al., 2015; Du et al., 2017; Payushina et al., 2018; Rong et al., 2019; Joseph et al., 2020). CM was collected from MSCs between passages 1 and 12 at 50–100% confluence. The most common model was murine (96.77%, 30/31), although a swine model was also used (Joseph et al., 2020). Regarding the route of administration, MSC-CM was mainly used topically (51.61%, 16/31), applied to the wounds or around them in cream, hydrogel, or membranes (Heo et al., 2011; Lee et al., 2011; Chen et al., 2014; Jun et al., 2014; Sun et al., 2014; Mehanna et al., 2015; Park et al., 2018; Joseph et al., 2020). MSC-CM was also injected (41.94%, 13/31), mainly subcutaneously (25.81%, 8/31) (Templin et al., 2009; Lee et al., 2011; Deng et al., 2017; He et al., 2020; Zhou et al., 2020) but also intradermally (Cho et al., 2010) and intraperitoneally (Fong et al., 2014). Subcutaneously injected and topically applied concomitant MSC-CM was also tested (Chen et al., 2008; Yoon et al., 2010). Topical application might be more effective than subcutaneous injections (Lee et al., 2011).

**TABLE 1:** Studies regarding mesenchymal stromal cell-conditioned medium for treating wounds in animal models.

	**MSC source**	**Method of tissue extraction**	**MSC characterization**	**Preparation of MSC-CM**	**Model**	**Groups of treatments and via of administration**	**Follow-up (days)**	**Assessment**	**Main outcome**	**Other outcomes**
Chen et al., 2021	Human subcutaneous adipose tissue samples	Liposuction	Flow cytometry (CD29+, CD90+, CD105+, CD31−, CD34−, CD45−). Osteogenic and adipogenic differentiation	MSCs of passage 3 at 90% confluence were used. CMs at different dilutions (100, 50, and 25%) were collected. Electrospun fibrous scaffolds formed by emulsion electrospinning; an ADSC-CM loaded micro-nano polylactic acid (PLA) electrospun fiber (MPF) was developed	Murine. Full-thickness excisional wounds, 15 mm × 15 mm, on dorsal surface	Wounds were covered with an electrospun membrane - Control - MPF alone - MPF loaded with 100% AT-MSC-CM - MPF loaded with 50% AT-MSC-CM - MPF loaded with 25% ATMSC-CM (*n* = 3/group)	15	Macroscopic appearance (photography), histology (HE, MT), IHC, qRT-PCR	MPF loaded with MSC-CM accelerated wound healing and prevented abnormal scar formation, promoting angiogenesis without adversely affecting epidermal cells	MPF loaded with MSC-CM inhibited ECM deposition, including Col I and Col III, and decreased α-SMA expression, showing an inhibitory effect on fibroblast differentiation. Groups with less collagen deposition and smaller scar area showed more VEGF expression and a faster healing rate. The wound area of MPF loaded with 100% AT-MSC-CM was only 34.9% of that of the control group
Zhou et al., 2020	Human dental pulp isolated from periodontally compromised teeth (P-DP-MSCs) and healthy teeth (H-DP-MSCs)	Teeth extracting	Flow cytometry (CD90+, CD105+, CD146+, CD31−, CD34−, CD45−). Osteogenic, adipogenic, and chondrogenic differentiation	MSCs of passage 4 at 90% confluence were used. CM was collected and, it was used to obtain EVs	Murine. Full-thickness excisional wounds, on the dorsal surface	100 μl subcutaneously injected at 4 sites around the wound (25 μl per site) - Control - P-DP-MSC-CM EVs - H-DP-MSC- CM EVs (*n* = 10/group)	14	Macroscopic appearance (photography), microscopic appearance (newly formed vessels), histology (HE), IHC (VEGF, CD31)	Wound closure was markedly accelerated by DP-MSC-CM EVs and P-DP-MSC-CM EVs. P-DP-MSC-CM EV group closed faster than H-DP-MSC-CM EV group (*p* < 0.05)	DP-MSC-CM extracellular vesicle-treated wounds had a lower level of scar formation and enhanced vessel formation in the wound sites. No adverse events were observed
Sabzevari et al., 2020	Wharton’s jelly from human umbilical cords	Cesarean section	Flow cytometry (CD44+, CD73+, CD90+, CD105+, CD14−, CD31−, CD34−, CD45−, HLA-DR−). Osteogenic, adipogenic, and chondrogenic differentiation	MSC of passages 4–6 at 70% confluence were used. WJ-MSCs were transfected with a recombinant construct encoding hCAP-18/LL-37 gene. Next, the CM of the transfected cells (LL-37-MSCs) was harvested, and its concentrate was formulated in a sodium alginate (SA)/gelatine (G) hydrogel	Murine. Full-thickness circular excision wound, 20 mm, below the skull	The hydrogel was placed on the wound and covered by a wound dressing - PBS (control) - SA/G-PBS group (the SA/G-2/8 hydrogel contained only PBS) - SA/G-V-CM group (the SA/G-2/8 hydrogel contained only CM vehicle) - SA/G-LL-37-CM group (the SA/G-2/8 hydrogel contained LL-37-CM) (*n* = 3/group)	21	Macroscopic appearance (photography), histology (HE, MT), qRT-PCR	WJ-MSC-CM hydrogel effectively accelerated wound contraction and promoted wound healing, even more in the SA/G-LL-37-CM group	Blood vessels were higher in SA/G-LL-37-CM group, showing the early angiogenesis profile with higher VEGF-A levels. This group also showed the best collagen arrangement, thickness, and density
Joseph et al., 2020	Caprine, canine, and guinea pig bone marrow	Aspiration needle from the iliac crest	Flow cytometry (CD 73+, CD90+, CD 105+, CD34−). Chondrogenic, osteogenic, and adipogenic differentiation	MSCs of passage 3 at 80% confluence were used. CM was collected and then lyophilized by the freeze−drying method and was vacuum sealed and stored at 4°C. A serum−free DMEM medium was processed for use as a control	Guinea pig. Full-thickness excisional skin wounds, 20 mm × 20 mm, on the dorsum on either side of the midline	100 μl of formulated respective CM was applied topically with sterile applicator over the wounds one per week - Control (laminin gel+DMEM) - Canine CM (laminin gel+canine MSC−CM) - Caprine CM (laminin gel+caprine MSC−CM)	28	Macroscopic appearance (photography), histology (HE)	MSC−CM accelerates excision wound healing compared with control (*p* < 0.05)	Surface epithelium, neovascularization, and collagen depositions improved more in MSC-CM-treated group than in controls (*p* < 0.05). Allogeneic and xenogenic application of CM significantly improved wound healing quality, with minimal scar formation. Better healing rate was observed in the allogeneic group
						- Guinea pig CM (laminin gel+guinea pig MSC−CM) (*n* = 6/group)				
He et al., 2020	Human amnion	Amniocentesis	Flow cytometry (CD73, CD45, CD31, CD105, CD44, CD34, CD90, CD29, SSEA-3, SSEA-4, EP-CAM, HLA-DR). Chondrogenic, osteogenic, and adipogenic differentiation	MSCs of passage 2 at 90% confluence were used. CM was collected	Murine. Full-thickness excisional skin wounds, 10 mm, on the dorsum	50 μl subcutaneous injected into the surrounding tissues of the wound bed at four sites - PBS (control) - MSC-CM - Murine LOXL2 (5 μg, Sino Biologicals, China) (*n* = 4/group)	12	Macroscopic appearance (photography), histology (HE, TC)	Wound sizes were significantly reduced in the LOXL2 and MSC-CM groups compared with controls (*p* < 0.05)	MSC-CM and LOXL2 enhanced wound healing. Epidermis of the MSC-CM and LOXL2 group mice resembled normal skin and the keratinocytes were well organized and tightly arranged. These treatments also significantly reduced fibrosis and improved keratinocyte proliferation. No adverse events were reported
Ahangar et al., 2020	Human bone marrow	Aspiration	−	MSCs of passage 1 at 70% confluence were used. CM was collected	Murine. Full-thickness excisional skin wounds, 6 mm^2^, on dorsal surface	100 μl was injected at four sites into the margins of each wound MSC-CM - DMEM (*n* = 8/group)	14	Macroscopic appearance (photography), histology (HE, TC), IHC, qRT-PCR	MSC-CM improved wound healing, showing a significant reduction in average wound area and wound width compared with DMEM-treated group (*p* < 0.05)	At day 7 post-wounding, wounds in MSC-CM-treated mice were 90% re-epithelialized compared with 78% in control group. MSC-CM also decreased inflammatory response, increased endothelial cell number and angiogenesis (showed in an increased numbers of CD31-positive
										endothelial cells), and increased both collagen I and III expression
Rong et al., 2019	ASC: sticks of antlers from sika deer hU-MSC: human umbilical cords	hU-MSC: Cesarean sections	−	MSCs of passage 3 at 80% confluence were used. CM was collected	Murine. Full-thickness skin excisional wounds, 8 mm in diameter, on the dorsal surface	MSC-CM was mixed with the hydrogel. 200 μl of each group was pipetted onto the wound every 2 days - DMEM - EGF - MSC-CM - ASC-CM (*n* = 8/group)	16	Photography, histology (HE), IHC	ASC-CM group accelerated wound healing compared with other treatments. At 16 days, wounds in ASC-CM group were completely closed, while other groups showed different sizes of unhealed wounds (DMEM, 6.14 ± 4.1 mm^2^; EGF, 1.79 ± 3.2 mm^2^; hU-MSC-CM, 0.61 ± 2.3 mm^2^, *p* < 0.05)	ASC-CM group had the thickest dermis containing the highest number of cutaneous appendages and the highest vessel numbers. The ASC-CM treatment significantly upregulated the expression ratios of Col3A1/Col1A2, TGF-β3/TGF-β1, MMP1/TIMP1 and MMP3/TIMP1
Robert et al., 2019	Human skin	Face-lifting	−	MSCs of passage 6 at 90% confluence were used. CM was collected	Murine. Full-thickness excisional wound, 6 mm in diameter, on dorsum surface	CM was directly applied to the wounds or embedded within hydrogels - Control, no treatment (*n* = 12) - Carrageenan hydrogel (*n* = 9) - Carrageenan hydrogel embedded with MSC-CM (*n* = 10) - Polyvinyl alcohol hydrogel (*n* = 10) - Polyvinyl alcohol hydrogel-embedded with MSC-CM (*n* = 11) - Only MSC-CM (*n* = 8)	14	Macroscopic appearance (photography), histology (HE)	All groups showed successfully repaired and closed wounds, although the animals treated with CM embedded in PVA (or only with PVA) displayed slightly larger wounds (*p* > 0.05)	Improvements in wound closure were not observed, but MSC-CM increased angiogenesis, independently of the hydrogel used
Raj et al., 2019	Human umbilical cord	Umbilical cord dissection	Flow cytometry (CD13+, CD29+, CD44+, CD90+, CD10−, CD14−, CD34−, CD117−)	MSCs were transduced with a lentiviral vector (green fluorescence protein tagged). MSCs of passage 3–4 were used. CM was collected. Wound dressing patches: impregnated of aloe verapolycaprolactone (AV/PCL) nanoscaffolds with hWJSCs or hWJSC−CM were also created	Murine. Full-thickness excisional wound, 8 mm, on the back	100 μl of: - PBS with 1 × 10^6^ MSCs - MSC-CM - PBS with 1 × 10^6^ fibroblast - Fibroblast-CM - UCM - PBS with 1 × 10^6^ MSCs+AV/PCL - MSC-CM+AV/PCL - PBS with 1 × 10^6^ fibroblast + AV/PCL - Fibroblast-CM+AV/PCL - PBS+AV/PCL - Untreated group (*n* = 9/group)	28	Macroscopic appearance (photography), histology (HE), IHC, qRT−PCR	The dermal thickness of both MSCs+AV/PCL (290.55 μm) and MSC−CM+AV/PCL (338.3 μm) treatment groups was significantly greater than that of the controls (PBS+AV/PCL, 193.51 μm; UCM, 266.55 μm; fibroblast+AV/PCL, 235.29 μm; fibroblast−CM+ AV/PCL, 227.31 μm)	CD31 marker showed strong positive signals in the MSC-CM group and in the MSCs group compared with untreated controls
Im et al., 2019	Human (type is not specified)	Purchased from Lonza (Basel, Switzerland)	−	MSCs of passages 6–12 were used. CM from the MSCs treated with or without gold–iron nanoparticles was collected	Murine. Full-thickness excisional wound, 20 mm × 20 mm, on the back	Daily injection of CM (200 μl/wound) was for 4 days - MSC-CM passage 6 - MSC-CM passage 12 - MSC-CM passage 12 with gold–iron nanoparticles (*n* = 4/group)	14	Macroscopic appearance (photography), histology (HE), IHC, qRT-PCR	All wounds were almost closed with similar appearance in all groups	Increased CD31 expression was observed in MSC- CM passage 12 with gold–iron nanoparticles group compared with the passage 12 without gold–iron nano- particles. SM-α expression did not exhibit differences between groups. The relative amount of involucrin and laminin was higher in MSC-CM passage 6 and MSC-CM passage 12 with gold–iron nanoparticles compared with MSC-CM passage 12
Yuan et al., 2018	Human adipose tissue	−	Flow cytometry (CD49+, CD73+, CD90+, CD105+, CD34−). Adipogenic and chondrogenic differentiation	MSCs of passages 2–5 were used. Cells were cultured in the presence of the profibrogenic cytokine TGF-β1. CM was collected	Murine. Full-thickness skin wounds, 6 mm punch, on the back	Wounds were applied with 0, 10, 50, and 100% MSC-CM* or left untreated (*n* = 7/group)	14	Macroscopic appearance (photography)	MSC-CM treatment significantly accelerated wound healing (*p* < 0.05)	Compared with the non-treated wounds and 0% MSC-CM group, 100% MSC-CM treatment accelerated wound healing on the seventh day after wounding (ratio of untreated wound area: 100% in untreated group, 100% in 0% MSC-CM, and 50% in 100% MSC-CM)
Payushina et al., 2018	Murine bone marrow from tibia and femur	−	Flow cytometry (CD73+, CD90+). Osteogenic and adipogenic differentiation	MSCs of passage 2 were used. CM was collected	Murine. Full-thickness skin wounds, 20 mm in diameter, on the back	100 μl MSC-CM or control CM was injected into the wound bed each other day five times (*n* = not specified)	14	Clinical examination, histology	MSC-CM group showed greater number of blood vessels (53.20 ± 2.58 blood vessels per field of view) compared with controls (37.20 ± 4.73), *p* < 0.05	MSC-CM group showed less intensive inflammation and more complete epithelialization compared with controls
Park et al., 2018	Human amniotic fluid	Amniocentesis	Flow cytometry (CD13+, CD29+, CD44+, CD71+, CD90+, CD120a+, CD31−, CD106−, CD15−, CD33−, CD34−, CD45−). Adipogenic, osteogenic, and chondrogenic differentiation	MSCs of passage 12 at 70–100% confluence were used. They were supplemented with selenium and bFGF. CM was collected	Murine. Full-thickness skin wounds, 8 mm, side of the midline	Vehicle, CM supplemented with selenium (−/s), CM supplemented with bFGF (b/−), or CM supplemented with bot selenium and bFGF (b/s), was topically applied to the induced wounds in a volume of 20 μl (*n* = 10/group)	11	Macroscopic appearance (photography), histology (HE, 3,3′-diaminobenzidine tetrahydrochloride staining), IHC	Treatment with CM (b/s) resulted in complete wound closure, while not completed closure was observed in the other groups	The CM (−/s) group exhibited better recovery than the CM (b/−) group. The CM (b/s) group showed the thickest epidermis region and the highest expression of involucrin. Smad2, AKT-MEK1/2-ERK, and NFκB signaling pathways were more effectively activated by CM (−/s) than by CM (b/−), and their
										highest activation was seen when treated with CM (b/s)
Tarcisia et al., 2017	Adipose tissue	−	Flow cytometry positive for (CD73+, CD90+, CD34−). Osteogenic, chondrogenic, and adipogenic differentiation	MSCs of passage 3 were used. CM was then collected	Murine. Full-thickness excisional skin wounds, 20 mm long and 5 mm depth, on the back	The four cuts of each rat were treated randomly with MSC-CM (100%), complete culture medium, basal medium, and without treatment (control). The treatment was only done once after the rat skin injury (*n* = 30/group)	28	Macroscopic appearance (photography), histology (HE, MT).	Wounds treated with MSC-CM showed improvement in wound healing process	MSC-CM showed greater collagen density, angiogenesis, ratio, and length of epithelialization than the other groups (*p* < 0.05)
Tarcisia et al., 2017	Adipose tissue	−	Flow cytometry positive for (CD73+, CD90+, CD34−). Osteogenic, chondrogenic, and adipogenic differentiation	MSCs of passage 3 were used. CM was then collected	Murine. Full-thickness excisional skin wounds, 20 mm long and 5 mm depth, on the back	The four cuts of each rat were treated randomly with MSC-CM (100%), complete culture medium, basal medium, and without treatment (control). The treatment was only done once after the rat skin injury (*n* = 30/group)	28	Macroscopic appearance (photography), histology (HE, MT).	Wounds treated with MSC-CM showed improvement in wound healing process	MSC-CM showed greater collagen density, angiogenesis, ratio, and length of epithelialization than the other groups (*p* < 0.05)
Lee et al., 2017	Adipose tissue	−	−	Ell3 expression was suppressed using siRNA transfection in MSCs. CM harvested from MSCs transfected with siNS or siEll3 was collected	Murine. Full-thickness excisional skin wound, back	100 μl of CM prepared from siNS- or siEll3-transfected MSCs or serum-free media was applied to the wound (*n* = not specified)	7	Macroscopic appearance (photography), histology (HE), IHC.	Skin wounds treated with siEll3 CM recovered to a lesser extent than those treated with serum-free media	siEll3 CM could not enhance the wound healing rate, whereas siNS CM significantly promoted wound repair
Du et al., 2017	Rabbit bone marrow	−	−	MSCs of passage 2 were used. MSCs were cultured in normoxic (Nc) and chemical hypoxic conditioning by adding CoCl_2_ (Cc). Decellularization was conducted and extracellular matrices (ECM) cell-free were constructed	Murine. Full-thickness excisional skin wound, 7 mm, on the back	No treated group (control), mice treated with Nc-ECM sheets or Cc-ECM sheets (*n* = 16/group)	7	Macroscopic appearance (photography), histology (HE, MT, picrosirius red)	All the Cc-ECM-treated wounds completely healed on day 7, while Nc-ECM-treated wounds healed about 85.0 ± 8.6%, and non-treated wounds only healed 69.8 ± 9.6%	No inflammatory signs or visible indication of necrosis or other adverse events were observed
Dong et al., 2017	Human umbilical cord	−	−	MSCs were transduced with human Wnt7a cDNA retroviral vector. MSCs of passage 3 at 50–60% confluence were used. Passage-3 MSCs were. CM from MSCs (MSC-CM) and from MSC with Wnt7a (Wnt-CM) was collected	Murine. Full-thickness excisional wound, 10 mm, on the back	100 μl of Wnt-CM, MSC-CM, or DMEM was subcutaneously injected at multiple points into the wound area (*n* = 3/group)	14	Macroscopic appearance (photography), histology (HE, MT)	Wnt-CM significantly enhanced the closure rates in comparison with MSC-CM and DMEM (91.5 vs. 76.3 vs. 65.1%, respectively)	Wnt-CM accelerated migration of HFs into the wound area, had a thicker epidermis with more organized cell layers, and showed regeneration of more hair follicles. Increased expression of the α-SMA, collagen I, and collagen III was also observed in Wnt-CM group
Dong et al., 2017	Human adipose tissues	−	−	Coleman adipose tissue was mechanically emulsified to obtain ECM/SVF-gel. For SVF preparation, the Coleman adipose tissue was digested. Supernatant from ECM/SVF-gel (gel-CM), Coleman adipose tissue (adi-CM), and SVF (SVF-CM) culture were collected to obtain CM	Murine. Full-thickness excisional skin wound, 8 mm, on the back	100 ml PBS (control), gel-CM, adi-CM, or SVF-CM was injected into the wounds (*n* = 15/group)	14	Macroscopic appearance (photography), histology (HE, MT), ELISA	CM treatments resulted in a significant upregulation of collagen production compared with controls, revealing that the Gel-CM-group had the highest production of collagen	Higher expression of bFGF, EGF, and TGF-b in Gel-CM was observed
Mehanna et al., 2015	Murine bone marrow	Needle flushing	Flow cytometry (CD44+, CD45−)	MSCs of passage 3 were used, and CM was collected	Murine. Full-thickness square-shaped skin wound (35 mm × 35 mm), on the back	Topical application - Untreated group (control) - Fibrin glue only group - Fibrin+MSCs group - Fibrin +MSC-CM group (*n* = 13/group)	35	Macroscopic appearance (photography), functional parameters (TEWL, SCH, tensile strength assessment), histology (HE, MT), IHC	Wounds’ size in fibrin+MSCs and fibrin+MSC-CM groups showed significant decrease in comparison with only fibrin and controls	CD68+ macrophages infiltrating granulation tissue were considerably higher in fibrin+MSC and fibrin+CM groups. SCH and tensile strength were higher, while TEWL was lower in both fibrin+MSC and fibrin+CM than in only fibrin and control group
Tam et al., 2014	Wharton’s jelly from human umbilical cord	Cutting umbilical cords	Flow cytometry (CD13+, CD29+, CD44+, CD90+, CD10−, CD14−, CD34, CD117)	MSCs of passage 3–4 were used. It was a constructed wound dressing patch made up of an aloe vera−PCL (AV/PCL) nanoscaffold impregnated with WJ-MSCs or its CM	Murine. Full-thickness skin wounds, 8 mm, on dorsal region	- MSCs+AV/PC - MSC-CM+AV/PCL - Fibroblast+ AV/PCL - Fibroblast-CM+AV/PCL - PBS+ AV/PCL - Untreated (*n* = 9/group)	28	Macroscopic appearance (photography), histology (HE and MT), IF, WB, qRT-PCR	MSCs+AV/PCL and MSC-CM+AV/PCL treatment arms showed faster wound closure compared with other groups (*p* < 0.05)	MSCs+AV/PCL and MSC-CM+AV/PCL groups showed higher numbers of sebaceous glands, hair follicles, cellularity, and vasculature compared with other groups
Sun et al., 2014	Murine abdominal subcutaneous adipose tissue	Incision	Flow cytometry (CD29+, CD90+, CD105+, CD34−). Osteogenic and adipogenic differentiation	MSCs of passage 3 at 90% confluence were used. Hypoxic microenvironment was generated using a disposable oxygen-absorbed and CO2 generator. CM was collected	Murine. Full-thickness skin incisions, 30 mm in diameter, on the back	Topical application - Concentrated hypoxic MSC-CM - Hypoxic MSC-CM - Serum medium (*n* = 10/group)	21	Macroscopic appearance (photography), histology (HE)	The average healing time was lower in concentrated hypoxic MSC-CM than in hypoxic MSC-CM and control group (16.2 ± 0.98 vs. 17.7 ± 0.78 vs. 21.3 ± 1.10 days)	Wound closure after treatment with concentrated hypoxic MSC-CM showed well-organized epidermis, thick cuticular layer, and increased collagen content than the other groups
Jun et al., 2014	Human amniotic fluid	Amniocentesis	Immunofluorescence. Adipogenic and osteogenic differentiation	MSCs at 70% confluence were used. Cells were cultured in normoxic (nor) and hypoxic (hypo) conditions. CM was collected	Murine. Full-thickness excisional skin wound, 2 mm full thickness, on each side of the midline	100 μl topically applied - MSC-hypoCM - MSC-norCM - DMEM (*n* = 10/group)	5	Macroscopic appearance (photography), histology (HE), IHC	MSC-hypoCM significantly accelerated wound closure compared with DMEM and MSC-norCM groups (*p* < 0.05)	The skin structure of wounds treated by MSC-hypoCM was more similar to normal skin structure. TGF-β/SMAD2 and PI3K/AKT signal pathways were upregulated in AF-MSC-hypoCM
Fong et al., 2014	Wharton’s jelly from human umbilical cord	Full-term delivery	Immunofluorescence (CD10+, CD13+, CD29+, CD44+, CD90+)	MSCs of passages 3–4 at 80% confluence were used. CM was then collected	Murine. Full-thickness excisional skin wound, 8 mm, on dorsum surface	100 μl injected intraperitoneally - MSCs - MSC-CM - Fibroblast - Fibroblast-CM - UCM (*n* = 9/group)	28	Macroscopic appearance (photography), histology (HE), IHC	MSC and MSC-CM healing rates were greater compared with controls (*p* < 0.05)	Wounds treated with MSCs and MSC-CMs showed greater re-epithelialization, vascularity, cellular density, sebaceous gland, and hair follicle numbers compared with controls
Chen et al., 2014	Human bone marrow	−	−	MSCs of passages 3–4 at 80% confluence were used. MSCs were cultured and expanded under normoxic or hypoxic conditions. CM was collected from the normoxic and hypoxic MSCs to yield norCM and hypoCM	Murine. Full-thickness excisional skin wounds, 18 mm, on dorsal surface	100 μl of treatment topically applied to skin wounds and covered with dressings daily for the first 4 days - MSC-norCM - MSC-hypoCM - Vehicle (control) (*n* = 16/group)	14	Macroscopic appearance (photography), IHC, IF	HypoCM-treated mice showed smaller wound area compared with norCM groups and vehicle control (26.42 ± 62.48 vs. 37.92 ± 62.44 vs. 45.00 ± 61.97%, respectively)	MSC-hypoCM accelerated wound closure compared with norCM and vehicle control
Tamari et al., 2013	Human bone marrow	−	−	MSCs purchased from LONZA	Murine. Full-thickness excisional skin wound, 8 mm, on the midline on the back	Injection administered at 4 spots around the wound - PBS - MSCs - MSC-CM (*n* = not specified)	14	Macroscopic appearance (photography), histology, ELISA	Epithelialization rate was higher in MSC and MSC-CM group compared with controls: non-epithelialized wound area after treatment was 42.64 ± 5.36% in the control group, 4.90 ± 2.36% in MSC group, and 5.74 ± 2.85% in MSC-CM group	MSC and MSC-CM accelerated wound healing. HA production in MSC and MSC-CM group was increased compared with controls
Lee et al., 2011	hCB: human cord blood hESC: human embryo	−	Fluorescence-activated cell sorter (CD133+, KDR+)	MSCs of passages 5–8 at 80 confluence were used. CM was collected	Murine. Full-thickness skin wound, 12 mm, on dorsal surface	200 μl of CM was subcutaneously injected around the wound site or applied topically - hESC-CM - hCB-CM - Control medium (*n* = 10/group)	21	Macroscopic appearance (photography), histology	Wound healing rate was higher in groups treated with CM (90, 70, and 40% in the hESC-CM, hCB-CM, and vehicle medium, respectively)	Wound closure rate in hESC-CM group was higher when it was used topically instead of subcutaneously
Heo et al., 2011	Human adipose tissue	Elective surgery	−	MSCs of passages 2–5 were used. CM was collected. Immunoprecipitation of TNF-α was also used, and CM implemented TNF-α was collected	Murine. Full-thickness excisional skin wounds, 8 mm, on the dorsal surface	20 μl topically applied on the wound bed daily - PBS - MSC-CM - MSC-CM with TNF-α (*n* = 8/group)	12	Macroscopic appearance (photography), histology	MSC-CM with TNF-α accelerated wound closure compared with PBS MSC-CM	Number of blood vessel was the highest in MSC-CM with TNF-α group
Yoon et al., 2010	Human amniotic fluid	Amniocentesis	Immunofluorescence (CD13+, CD29+, CD44+). Osteogenic, adipogenic, and chondrogenic differentiation	MSCs of passage 3 at 70% confluence were used. CM was collected	Murine. Full-thickness excisional skin wound, 2 mm, on each side of the midline.	100 μl subcutaneous injection around the wound and topically applied on the wound bed	8	Macroscopic appearance (photography), histology (HE), IHC	MSC-CM accelerated wound closure compared with control	No difference in skin structure was observed between groups
						- Control medium				
						- MSC-CM (*n* = 10/group)				
Cho et al., 2010	Human subcutaneous adipose tissue	Liposuction	Flow cytometry (CD90+, CD49d−)	MSCs of passage. TGF-β1-treated MSC-CM or non-treated MSC-CM was collected	Murine. Circular full-thickness skin wounds, 4-mm diameter, on the back	Intradermal injections (0.05 ml/point × 4 points) into the wound base twice per week	10	Macroscopic appearance (photography), histology (HE)	Wound size was reduced in both groups	TGF-β1-treated MSC-CM accelerated wound healing compared with MSC-CM
						- Bactroban oint with MSC-CM - Bactroban oint with TGF-β1-treated MSC-CM (*n* = 3/group)				
Templin et al., 2009	Murine bone marrow	−	−	Retroviral gene transfer into lin- cells was performed, and DK mix cells were created. Cells and CM were then collected	Murine. Full-thickness skin wounds, 5-mm diameter, on dorsal surface	200 μl subcutaneously injected around the wound at 8 different sites - MSC-CM - MSCs - PBS (*n* = 8/group)	13	Macroscopic appearance (photography),	MSC and MCS-CM accelerated wound healing compared with PBS (area non-epithelialized after follow-up: MSC = 25.9 ± 2.6%, MSC-CM = 25.1 ± 1.5%, PBS = 48.3 ± 4.7%)	Capillary density was higher in MSC-CM and MSC groups
Chen et al., 2008	Murine bone marrow from femurs and tibia	−	q	MSCs in passage 3 at 80% confluence under hypoxic conditions were used	Murine. Full-thickness skin wounds, 6-mm diameter, on each side of midline	100 ml (80 ml for subcutaneous injection around the wound and 20 ml for topical application on the wound bed) - MSC-CM - Fibroblast-CM - Vehicle (*n* = 5/group)	14	Macroscopic appearance (photography), histology (HE), IHC	MSC-CM significantly accelerated wound closure compared with fibroblast-CM or vehicle (wound closure percentage: 61 vs. 53 vs. 51%, respectively)	MSC-CM increased cell recruitment.

ASCs, antler stem cells; AT, Adipose tissue-derived; bFGF, basic fibroblast growth factor; AF, amniotic fluid; BM, bone marrow; CM, conditioned medium; DMEM, Dulbecco’s modified Eagle medium; DP, dental pulp; ECM, extracellular matrix; EGF, epidermal growth factor; ELISA, enzyme-linked immunosorbent assay; EVs, extracellular vesicles; Exos, exosomes; HE, hematoxylin and eosin; hCB, human cord blood; hESC, human embryonic stem cell; IHC, immunohistochemistry; IF, immunofluorescence; MPF, micro-nano polylactic acid electrospun fiber; MSCs, mesenchymal stromal cells; MT, Masson’s trichrome; PBM, photobiomodulation; PBS, phosphate-buffered saline; qRT-PCR, real-time quantitative polymerase chain reaction; SVF, stromal vascular fraction; UCM, unconditioned medium; WB, Western blotting; WJ, Wharton’s jelly. *0, 10, 50, and 100% MSC-CM refers to the dilutions of the conditioned medium in DMEM/F12 with 2% FBS.

MSC-CM was tested for treating wounds in 421 animals. The mean wound size was 11.65 mm (from 2 to 35 mm), and the mean follow-up was 16.48 days (from 5 to 35 days). Wounds were assessed mainly by macroscopic appearance, histology, immunohistochemistry, and qRT-PCR. Outcomes showed that MSC-CM had better results in terms of wound closure, re-epithelialization, and vascularization than non-treated groups or other treatment groups (phosphate-buffered saline (PBS), hydrogel without cells, Dulbecco’s modified Eagle medium (DMEM), and unconditioned medium). Moreover, some research compared the effects between MSCs and MSC-CM with similar results. Topically applied MSCs and their CM via fibrin vehicle showed similar wound healing rates (Mehanna et al., 2015). Healing rates, vascularity, and cellular density were similar between MSCs and MSC-CM injected intraperitoneally (Fong et al., 2014). Injections of MSCs or MSC-CM around the wound showed similar results in wound healing (Templin et al., 2009; Tamari et al., 2013) (non-epithelialized area after 14 days’ follow-up: 25.9 ± 2.6% in MSCs and 25.1 ± 1.5% in the MSC-CM group).

Different MSC sources and CM delivery were also compared. Allogeneic MSC-CM showed better healing rate than xenogeneic MSC-CM treatment (Joseph et al., 2020). The wound closure rate was higher in animals treated with carrageenan-embedded CM than mice treated with polyvinyl alcohol-embedded CM (Robert et al., 2019). Moreover, CM supplemented with selenium and basic fibroblast growth factor (bFGF) showed better results in wound healing than CM alone or CM only supplemented with bFGF or selenium, as complete wound closure after 11 days’ follow-up was only observed in CM supplemented with both growth factors (Park et al., 2018). Hypoxic conditions also improved wound healing, accelerating wound closure (Chen et al., 2014; Jun et al., 2014; Sun et al., 2014; Du et al., 2017). TNF-α- and TFG-β1-implemented CM also accelerated wound healing as compared with non-supplemented MSC-CM (Cho et al., 2010; Heo et al., 2011). CM derived from *Wnt7a*-transduced MSCs also showed higher closures rates than did the MSC-CM group (Dong et al., 2017). No adverse events were reported in these studies.

##### Diabetic wounds

Nineteen studies evaluated the effects of MSC-CM for treating diabetic wounds (Table 2). To obtain CM, cells were mainly isolated from human tissues (89.47%, 17/19): from umbilical cord blood (Kim et al., 2010; Kusindarta et al., 2016; Chen Z. et al., 2018; Raj et al., 2019; Zhang S. et al., 2020) (29.41%, 5/17), bone marrow (Pouriran et al., 2016; Amini et al., 2018; Bagheri et al., 2018; Saheli et al., 2020) (23.53%, 4/17), adipose tissue (Deng et al., 2019; De Gregorio et al., 2020) (11.76%, 2/17), and Wharton’s jelly (Fong et al., 2014; Tam et al., 2014) (11.76%, 2/17). Human hair follicles (Ma et al., 2015), menstrual blood (Dalirfardouei et al., 2019), and urine (Chen C. Y. et al., 2018) were also used. Moreover, murine (Li T. et al., 2019) and swine (Irons et al., 2018) adipose tissues were employed. CM was collected from MSCs between passages 1 and 12 at 50–100% confluence. The most common model was murine (94.12%, 16/17), although a swine model was also used (Irons et al., 2018). Regarding the route of administration, MSC-CM was injected (78.95%, 15/19) mainly intraperitoneally (26.31%, 5/19) (Fong et al., 2014; Pouriran et al., 2016; Amini et al., 2018; Bagheri et al., 2018; Saheli et al., 2020), subcutaneously (15.79%, 3/19) (Chen C. Y. et al., 2018; Li T. et al., 2019; Zhang S. et al., 2020), intradermally (15.79%, 3/19) (Kim et al., 2010; Dalirfardouei et al., 2019; Deng et al., 2019), or intravenously (5.26%, 1/19) (De Gregorio et al., 2020). MSC-CM was also applied topically (21.05%, 4/19) to the wounds or around them in creams, hydrogels, or membranes (Kusindarta et al., 2016; Chen Z. et al., 2018; Irons et al., 2018).

**TABLE 2:** Studies regarding mesenchymal stromal cell-conditioned medium for treating diabetic wounds in animal models.

	**MSC source**	**Method of tissue extraction**	**MSC characterization**	**Preparation of MSC-CM**	**Model**	**Groups of treatments and via of administration**	**Follow-up (days)**	**Assessment**	**Main outcome**	**Other outcomes**
Zhang S. et al., 2020	Human umbilical cords	Full-term delivered infants	Flow cytometry (CD105+, CD73+, CD90+, CD166+, CD54+, CD13+, CD45−, CD34−, CD14−, CD19−, CD117−, HLA-DR−)	MSCs of passages 3–4 at 80% confluence were used. CM was collected	Murine. Diabetic model. Full-thickness excisional skin wounds, 800 mm^2^, on the back	150 μl subcutaneously injected around the wounds at six injection sites (25 μl per site) every day for three consecutive days - Control (non-CM) - FB - UC-MSC - UC-MSC-CM (*n* = 12/group)	14	Macroscopic appearance (photography), histology (HE), IHC, qRT-PCR	UC-MSC and UC-MSC-CM treatment accelerated wound healing rate. The wound area of UC-MSC and UC-MSC-CM was ≈15% reduced compared with controls	UC-MSC and UC-MSC-CM increased the percentage of M2 macrophages in the local wounds and the levels of anti-inflammatory cytokines, IL-10, and VEGF and significantly decreased the levels of proinflammatory cytokines, IL-1β, TNF-α, and IL-6
Saheli et al., 2020	Human bone marrow	−	Flow cytometry (CD73+, CD90+, CD105+, CD34−, CD45−). Osteogenic and adipogenic differentiation	MSCs of passage 4 at 80% were used. CM was then collected	Murine. Diabetic model. Full-thickness excisional skin wounds, 20 mm long, on the chest proximal part intraperitoneal	50-fold concentrated DMEM or CM twice at 12 and 24 h after wounding intraperitoneally - Control (no treatment) - Placebo - hBM-MSC-CM (*n* = 6/group)	15	Macroscopic appearance, histology (HE, MT), qRT-PCR	MSC-CM accelerated diabetic wound closure (67% wound closure in CM group vs. 33% in placebo group, *p* < 0.05)	MSC-CM treatment leads to upregulation of EGF and bFGF genes and higher cell viability/proliferation and migration
De Gregorio et al., 2020	Human subcutaneous adipose tissue samples from abdominal region	Liposuction	Flow cytometry (CD29+, CD13+, CD105+, CD73+, CD90+, CD235a−, CD31−, CD45−). Adipogenic and osteogenic differentiation	MSCs of passage 3 at 70% confluence were used. Cells were supplemented with 400 μM DFX (preconditioned MSCs) or with saline (vehicle) as non-preconditioned MSCs. CM was then collected	Murine. Diabetic mice. Full-thickness skin (2.5 mm × 3.5 mm) surgically removed from the dorsal surface of both feet, mimicking a foot ulcer	Intravenous administration of 50 μl of CM every 2 weeks - CM derived from DFX-preconditioned MSCs - CM derived from non-preconditioned MSCs - Vehicle (*n* = 6/group)	14	Macroscopic appearance (photography), histology (HE, MT), qRT-PCR, proteomic analysis	MSC-CM accelerated wound healing. The wound area of MSC-CM was ≈20% reduced compared with controls at day 7	MSC-CM derived from DFX-preconditioned MSCs had a more potent effect in recovering the skin vasculature
Bian et al., 2020	Human placenta	Cesarean section births	Flow cytometry (CD73+, CD90+, CD105+, CD19−, CD34−, CD45−, HLA-DR−). Osteogenic, adipogenic, and chondrogenic differentiation	MSCs of passages 3–7 were used. CM was collected, and EVs were obtained	Murine. Diabetic model. Full-thickness excisional wounds, 16 mm, on the back	100 μl was injected around the wounds at 4 sites (25 μl per site) - MSC-EVs - PBS (*n* = 5/group)	28	Photography, histology (HE, MT), IHC	MSC-EVs significantly accelerated wound healing. The narrowest scar widths were observed at day 14 post-wounding (2.41 ± 0.24 mm in MSC-EVs group vs. 3.87 ± 0.60 mm in PBS group)	CXCR4, p21 PCNA, and α-SMA were upregulated in the MSC-EV group
Raj et al., 2019	Human umbilical cord	Umbilical cord dissection	Flow cytometry (CD13+, CD29+, CD44+, CD90+, CD10−, CD14−, CD34−, CD117−)	MSCs were transduced with a lentiviral vector (green fluorescence protein tagged). MSCs of passages 3–4 were used. CM was collected. Wound dressing patches: impregnated with aloe verapolycaprolactone (AV/PCL) nanoscaffolds with hWJSCs or hWJSC−CM were also created	Murine. Diabetic model. Full-thickness excisional wounds, 6 mm, on the back	100 μl of: - PBS with 1 × 10^6^ MSCs - MSC-CM - UCM - PBS with 1 × 10^6^ MSCs+AV/PCL - MSC-CM+AV/PCL - UCM+ AV/PCL - Untreated group (*n* = 12/group)	28	Macroscopic appearance (photography), histology (HE), IHC, qRT−PCR	Thickness of the epidermis and dermis was significantly greater in both MSCs and MSC-CMs without AV/PCL compared with their controls without AV/PCL	AV/PCL groups showed an earlier re-epithelialization and increases in thickness of dermis and epidermis, cellularity, vasculature, and hair follicle numbers
Li T. et al., 2019	Murine adipose tissue	−	Flow cytometry and oil red O staining (CD29+, CD90, CD45−)	MSCs of passages 3–5 were used. They were cultured on various matrices (tissue culture plates (TCP), pure three-dimensional-printed	Murine. Diabetic murine model. Full-thickness skin defects, 7 mm diameter	200 μl of each solution was used (150 μl injected subcutaneously around the defect and 50 μl smeared onto the wound bed) - DMEM (control)	14	Macroscopic appearance (photography), histology (HE, MT), IF	All groups improved wound healing. The highest wound-healing rate was observed in DOPA-BC-CM group. Remaining wound area at	The newly formed capillary network around the excisional regions was the most intense in the DOPA-BC-CM group. Higher levels of CD31 and a higher amount of
				bioceramic (BC), and polydopamine-modified BC scaffolds (DOPA-BC), and each CM was collected		- MSC-CMs derived from TCP - MSC-CMs derived from BC - MSC-CMs derived from DOPA-BC (*n* = 4/group)			day 14 was 7.1 ± 3.4% in DOPA-BC-CM group, ∼15.2 ± 6.6% in BC-CM, ∼21.2 ± 11.3% in TCP-CM, and ∼31.8 ± 7.2% in DMEM group	collagen deposition were also observed in this group
Deng et al., 2019	Human adipose tissue	Liposuction	−	SVF gel was prepared, and its CM was collected (Gel-CM). CM from MSCs was also collected (MSC-CM)	Murine. Diabetic model. Full- thickness excisional wound, 20-mm diameter, on the back	100 μl was administered intradermally every 2 days: - Gel-CM - MSC-CM - PBS (*n* = 18/group)	14	Macroscopic appearance (photography), histology (HE), ELISA	The wound size in all groups was reduced. Wound-healing rate in the Gel-CM-treated group was significantly higher than that in the MSC-CM group (*p* < 0.05)	Gel-CM-treated rats exhibited complete re-epithelialization of the wound, while MSC-CM did not. Number of capillaries in the Gel-CM-treated group was higher in MSC-CM
Dalirfardouei et al., 2019	Human menstrual blood	Collecting menstrual blood from healthy women	Flow cytometry (CD29+, CD44+, CD90+, CD34−, CD45−, CD117−, HLA−DR-). Adipogenic and osteogenic differentiation	MSCs of passages 4–6 at 70–80% confluence were used. CM was collected, and exosomes were isolated	Murine. Diabetic mice models. Full-thickness excisional wound including the panniculus carnosus, 8 mm, on the back	100 μl was intradermally injected around the wound - PBS - MSCs - Exos-MSCs (*n* = 6/group)	14	Macroscopic appearance (photography), histology (HE, MT), IHC, qRT-PCR	Increased wound closure was observed in Exo-group compared with the control or MSC group. At day 12, wound closure was 84.34 ± 7.00% in Exo-MSCs, 46.4 ± 8.5% in MSCs, and 43.78 ± 6.95% in controls	Microvessel density was significantly higher in the Exo-group compared with the other two groups. Size of scar tissues significantly decreased in themice treated with MSCs and their exosomes compared with control group. A major reduction in the granulation tissue cellularity was observed in mice treated with exosomes compared with the cell group
Irons et al., 2018	Adipose tissue from pigs’ gluteal regions	Wound incision	−	MSCs at 90% were used. CM was collected	Pigs. Diabetic pigs. Full-thickness skin wounds, 50 mm circular, on the back	-Injection of low-dose MSCs - Injection of high-dose MSCs - Injection of low-dose EC/MSCs - Injection of high-dose EC/MSCs - 2 ml of MSC-CM topically applied every 3 days - 2 ml of EC-CM topically applied every 3 days - 2 ml of serum-free medium topically applied every 3 days (control) (*n* = 7/group)	28	Macroscopic appearance (photography), histology (HE)	Wounds treated with MSCs and MSC-CMs displayed a significant increase in the percentage of wound closure compared with controls (*p* < 0.05)	Decreases in the acute inflammation scores were observed in wounds treated with MSCs and MSC-CMs compared with controls
Chen Z. et al., 2018	Human umbilical cord	Umbilical cords sections	Electron microscopy (CD63+, CD81+)	MSCs of passages 3–5 at 100% confluence were used. CM was collected, and Exos were obtained. PF-127 hydrogel was mixed with Exos, and PF-127 composite (MSC-Exos/PF-127) was obtained	Murine. Diabetic rat model. Full-thickness skin wounds, 10 mm circular, on the back	Treatment was used topically - 100 μg MSC-Exos dissolved in 100 μl Pluronic F127 hydrogel (24%) - 100 μg MSC-Exos dissolved in 100 μl PBS - 100 μl PF-127 hydrogel (24%) - 100 μl PBS (control) (*n* = 6/group)	14	Macroscopic appearance (photography), histology (HE), IHC, qRT-PCR, IF	Wound area was significantly smaller in the MSC-Exos/PF-127 group than in the other groups. Wounds in the MSC-Exos/PF-127 group were almost completely healed at day 14, while the wound healing rates in the MSC-Exos, PF-127 hydrogel, and control groups were 8.95, 14.52, and 23.09%, respectively	New hair was only evident in the MSC-Exos/PF-127 group. Number of blood vessels was higher in the MSC-Exos/PF-127 and MSC-Exos groups than in the PF-127 hydrogel or control group
Chen C. Y. et al., 2018	Human urine samples	Urine sample collection	Flow cytometry, and electron microscopy (CD29+, CD44+, CD73+, CD90+, CD34−, CD45−). Osteogenic, adipogenic, and chondrogenic differentiation	MSCs of passages 2–6 at 80–90% confluence were used. CM was collected, and Exos were created. Lentivirus shRNAs were transfected	Murine. Diabetic rat model. Full-thickness skin wounds, 6 mm, on upper back	Treatment was subcutaneously injected around the wounds at 4 injection sites (25 μl per site) - 100 μl PBS - 200 μg MSC-Exos in 100 μl PBS - 200 μg MSC-Exos without DMBT1 in 100 μl PBS (*n* = 8/group)	12	Macroscopic appearance (photography), histology (HE, MT), IHC, qRT-PCR, IF	Faster wound closure was observed in MSC-Exos group compared with controls and MSC-Exos without DMBT1	Higher rate of re-epithelialization, lower level of scar formation, and higher number of newly formed blood vessels were observed in MSC-Exos group compared with controls and MSC-Exos without DMBT1
Bagheri et al., 2018	Human bone marrow	Aspiration	Flow cytometry (CD73+, CD90+, CD105+, CD34−, CD45−)	MSCs of passage 4 at 80% confluence were used. CM was collected	Murine. Diabetic rat model. Full-thickness skin wounds, 12 mm, on upper back	PBM was administered once daily, 6 days per week. CM was administered at days 0 and 1 intraperitoneally - DMEM vehicle (control) - MSC-CM - PBM - PMB+MSC-CM (*n* = 18/group)	15	Stereological methods, tensiometric examination	MSC-CM and PBM+MSC-CM increased the tensiometric properties compared with DMEM and PBM	MSC-CM, PBM, and PBM+MSC-CM groups showed a significant decrease in the three types of mast cells and in the total number of mast cells compared with controls
Amini et al., 2018	Human bone marrow	Aspiration	Flow cytometry (CD73+, CD90+, CD105+, CD34−, CD45−)	MSCs of passage 4 at 80% confluence were used. CM was collected	Murine. Diabetic rat model. Full-thickness skin wounds, 12 mm, on upper thoracic and lumbar regions	PBM was administered once daily, 6 days per week. CM was administered at days 0 and 1 intraperitoneally - DMEM vehicle (control) - MSC-CM - PBM - PMB+MSC-CM (*n* = 18/group)	15	Stereological methods, tensiometric examination, qRT-PCR	All treated groups significantly enhanced wound healing compared with controls. The extent of healing was significantly greater in the CM+PBM group	Number of fibroblast and epidermal cells, the lengths of blood vessels, and bFGF and SDF-1α expression were significantly higher in the CM+PBM group
Pouriran et al., 2016	Human bone marrow	Aspiration	Flow cytometry (CD105+, CD90+, CD73+, CD34−, CD45−)	MSCs of passage 4 at 80% confluence were used. CM was then collected	Murine. Diabetic rat model. Full-thickness skin wounds, 12 mm, on the thoracic and lumbar regions	PWLLLT was administered once daily, 6 days per week. MSC-CM was administered twice intraperitoneally - Non-treated - MSC-CM - PWLLLT - MSC-CM+PWLLLT (*n* = 7/group) - Cream containing 1 ml MSC-CM in ratio 10 g cream base - Povidone iodine (control) (*n* = 6/group)	15	Macroscopic appearance (photography), biomechanical examination	PWLLLT and MSC-CM, alone or in combination, improved biomechanical parameters in the wound	PWLLLT was more effective compared with MSC-CM
Kusindarta et al., 2016	Human umbilical cord	−	−	MSCs of passage 4 at 60% confluence were used. CM was then collected	Murine. Diabetic rat model. Full-thickness skin wounds, 7 mm, on the left side of the body	Topical application twice daily	9	Macroscopic appearance (photography), histology (HE)	MSC-CM induced faster re-epithelialization than other groups	MSC-CM promoted increasing density of collagen fiber and stimulated hair follicle and muscle regeneration greater than the other groups
Ma et al., 2015	Human hair follicle	Dissection from excess scalp tissue discarded after surgery	Flow cytometry (CD105+, CD29+, CD49b+, CD49d+, CD73+, CD271+, GD2+, CD90−, CD44−, CD34−, CD45−). Adipogenic, osteogenic and chondrogenic differentiation	MSCs of passage 1 at 80–90% confluence were used. CM was then collected	Murine. Diabetic rat model. Full-thickness skin wound, 6 mm, on dorsal surface	100 ml injected into each wound - DMEM - Normal fibroblast-CM - HF-MSC-CM (*n* = 3/group)	24	Macroscopic appearance (photography), histology (HE)	HF-MSC-CM accelerated wound healing compared with the other groups. The average number of days to complete wound closure in the group administered with HF-MSC-CM was 18.7 days compared with	The epidermal thickness of the HF-MSC-CM-treated wound sites was significantly higher than the other groups
									22.3 days in the group treated with fibroblast-CM and 24 days in DMEM group	
Tam et al., 2014	Wharton’s jelly from human umbilical cord	Cutting umbilical cords	Flow cytometry (CD13+, CD29+, CD44+, CD90+, CD10−, CD14−, CD34, CD117)	MSCs of passage 3–4 were used. It was constructed wound dressing patch made up of an aloe vera−PCL (AV/PCL) nanoscaffold impregnated with WJ-MSCs or its CM	Murine. Full-thickness skin wounds, 6 mm, on dorsal region	- MSCs+AV/PCL - MSC-CM+AV/PCL - UCM + AV/PCL (*n* = 12/group)	28	Macroscopic appearance (photography), histology (HE and MT), IF, WB, qRT-PCR	MSCs+AV/PCL and MSC-CM+AV/PCL groups showed faster wound closure compared with other groups	MSCs+AV/PCL and MSC-CM+AV/PCL groups showed increased numbers of sebaceous glands and hair follicles and greater cellularity and vasculature compared with other groups
Fong et al., 2014	Wharton’s jelly from human umbilical cord	Full-term delivery	Immunofluorescence (CD10+, CD13+, CD29+, CD44+, CD90+)	MSCs of passages 3–4 at 80% confluence were used. CM was then collected	Murine. Full-thickness excisional skin wound, 6 mm, on dorsum	100 μl injected intraperitoneally - MSCs - MSC-CM - UCM (*n* = 12/group)	28	Macroscopic appearance (photography), histology (HE), IHC	MSC and MSC-CM healing rates were greater compared with controls	MSCs and MSC-CMs showed greater re-epithelialization, increased vascularity, cellular density, sebaceous gland, and hair follicle numbers compared with controls
Kim et al., 2010	Human umbilical cord		Not specified (CD34+, CD31+, KDR+, Tie2+)	Cells were cultured, and CM was then collected	Murine. Diabetic rats. Full-thickness excisional wounds, 5 mm, on dorso-lateral area	Intradermal injections injected at three different intact dermis site near the wound - MSC - MSC-CM - PBS (*n* = 6/group)	12	Macroscopic appearance (photography), histology (HE)	MSC-CM and MSC promoted wound healing greater than controls	MSC-CM and MSC groups showed greater increases in neovascularization compared with controls. The effect of MSC and MSC-CM improving wound healing was similar

*AT, adipose tissue-derived; bFGF, basic fibroblast growth factor; AF, amniotic fluid; BM, bone marrow; CM, conditioned medium; DFX, deferoxamine; DMEM, Dulbecco’s modified Eagle medium; DP, dental pulp; EC, endothelial-differentiated; ECM, extracellular matrix; EGF, epidermal growth factor; ELISA, enzyme-linked immunosorbent assay; EVs, extracellular vesicles; Exos; exosomes; HE, hematoxylin and eosin; hCB, human cord blood; hESC, human embryonic stem cell; IHC, immunohistochemistry; IF, immunofluorescence; MPF, micro-nano polylactic acid electrospun fiber; MSCs, mesenchymal stromal cells; MT, Masson’s trichrome; PBM, photobiomodulation; PBS, phosphate-buffered saline; qRT-PCR, real-time quantitative polymerase chain reaction; SVF, stromal vascular fraction; UCM, unconditioned medium; WB, Western blotting; WJ, Wharton’s jelly.*

MSC-CM has been tested on 256 animals with diabetic wounds. The mean wound size was 13.47 mm (from 3.5 to 50 mm), and the mean follow-up was 18.83 days (from 9 to 28 days). Wounds were assessed mainly by macroscopic appearance, histology, immunohistochemistry, and qRT-PCR. MSC-CM had better results in terms of wound closure, re-epithelialization, and vessel formation than non-treated groups or other treatment groups (PBS, hydrogel without cells, DMEM, unconditioned medium, or povidone iodine). Moreover, some studies compared the effect between cells and its CM with similar results (Kim et al., 2010; Fong et al., 2014; Tam et al., 2014; Irons et al., 2018; Dalirfardouei et al., 2019; Zhang S. et al., 2020). MSC-CM was also used to heal wounds in combination with laser therapy, showing that the combined treatment enhanced wound healing compared with laser or MSC-CM alone (Pouriran et al., 2016; Amini et al., 2018; Bagheri et al., 2018).

Different ways of CM implementation have been also evaluated. CM supplemented with deferoxamine, a hypoxic mimetic agent, had a more potent effect on recovering skin vasculatures than non-supplemented CM (De Gregorio et al., 2020). DMBT1 expression also improves wound healing (Chen C. Y. et al., 2018). Moreover, CM cultured on polydopamine-modified three-dimensional-printed bioceramic (DOPA-BC) scaffolds showed a higher healing rate than CM cultured on tissue culture plates (TCPs) or pure three-dimensional-printed bioceramic (pure BC) (Li T. et al., 2019). After 14 days’ follow-up, the remaining wound area was 7.1 ± 3.4% in the DOPA-BC-CM group, 15.2 ± 6.6% in the BC-CM group, and 21.2 ± 11.3% in TCP-CM group (Li T. et al., 2019).

##### Other wounds

MSC-CM was also evaluated in second- and third-degree burn wounds (Aryan et al., 2019; Li J. Y. et al., 2019; Zhou et al., 2019), radiation-induced wounds (Sun et al., 2019), and infected wounds (Fridoni et al., 2019; Kouhkheil et al., 2019; Table 3). Cells were isolated from human tissues: bone marrow (Aryan et al., 2019; Fridoni et al., 2019; Kouhkheil et al., 2019), umbilical cord (Zhou et al., 2019), Wharton’s jelly (Sun et al., 2019), and placenta (Li J. Y. et al., 2019). MSCs from passages 3 to 8 at 60–80% confluence were used. All tested models were murine.

**TABLE 3:** Studies regarding mesenchymal stromal cell-conditioned medium for treating other types of wounds in animal models.

	**MSC source**	**Method of tissue extraction**	**MSC characterization**	**Preparation of MSC-CM**	**Model**	**Groups of treatments and via of administration**	**Follow-up (days)**	**Assessment**	**Main outcomes**	**Other outcomes**
Zhou et al., 2019	Human umbilical cords	Umbilical cord section from full-term healthy fetuses that were born via cesarean section	Flow cytometry (CD29+, CD44+, CD73+, CD90+, CD105+, CD34−, CD45, CD31−, CD271−)	MSCs of passages 3–8 at 70–80% confluence were used. CM was collected. The thermosensitive MSC-CM/hydrogel was prepared by mixing the precooled MSC-CM, chitosan/β-GP, and collagen solutions at a ratio of 1:2:1 on ice	Murine. Third-degree burned mice, 15 mm	Wound was covered and changed twice daily - Unconditioned medium (UM) - MSC-CM group - UM/hydrogel - MSC-CM/hydrogel (*n* = 18/group)	28	Macroscopic appearance (photography), histology (HE), IHC	Application of the MSC-CM/hydrogel shortened healing time. The average healing time in MSC-CM/hydrogel group was approximately 5 days shorter than in UM group and shorter than MSC-CM and UM/hydrogel groups	MSC-CM/hydrogel limited the area of inflammation; enhanced re-epithelialization; promoted the formation of high-quality, well-vascularized granulation tissue; and attenuated the formation of fibrotic and hypertrophic scar tissue
Sun et al., 2019	Wharton’s jelly from human umbilical cords	Section from umbilical cords	−	MSCs of passage 3 at 60% confluence were used. CM was collected	Murine. Radiation-induced skin injury rat model, 20 mm × 20 mm	200 ml of hydrogel was pipetted onto the radiation wound every 2 days - Non-treated - EGF - MSC-CM (*n* = 12/group)	56	Macroscopic appearance (photography), histology (HE), IHC	MSC-CM significantly accelerated wound closure and enhanced the wound healing quality. The great difference was observed at day 42, with a relative wound size of the MSC-CM group 2.63-fold smaller than the EGF group and 3.38-fold smaller than the non-treated group (0.8 ± 0.29, 2.1 ± 0.37, 2.7 ± 0.34 mm^2^, respectively)	α-SMA, Ki-67 expression, and the number of vessels/HPF were increased in MSC-CM group
Li J. Y. et al., 2019	Human fetal placenta	Placenta section	Flow cytometry adipogenic and qRT-PCR (CD29+, CD73+, CD105+, CD90+, CD34−, CD45−, CD133−). Adipogenic and osteogenic differentiation	MSCs of passages 3–7 at 80% confluence were used. CM was collected	Murine. Second-degree burn injury model. Back skin of mice was injured with 80°C water for 100 s to create a 10-mm diameter wound	200 μl of the treatments was subcutaneously injected near the wound at four sites - PBS containing 2 × 10^6^ MSCs - MSC-CM - PBS - DMEM (*n* = 5/group)	21	Macroscopic appearance (photography), histology (HE), IF	MSCs and MSC-CMs promoted wound healing compared with PBS and DMEM	High levels of new blood vessels and tubular structures were observed in the MSC and MSC-CM groups
Kouhkheil et al., 2019	Human bone marrow	Aspiration	Flow cytometry	MSCs of passage 4 at 80% confluence were used. CM was collected	Murine. MRSA rats infected. Full-thickness excisional wound, 15 mm, on the back	PBM was administered once daily, 6 days per week. 50 μl of the 10-fold CM was administered from day 0 until day 3 - Control - PBM - MSC-CM - PBM+MSC-CM (*n* = 18/group)	15	Clinical observation, microbiological, tensiometric, and stereological analyses	There was a significant decrease in colony-forming units in PBM+MSC-CM and PBM groups compared with controls	PBM+MSC-CM, PBM, and MSC-CM groups significantly increased wound strength compared with the control group. The PBM+MSC-CM and PBM groups had more stable MCs, less significant degranulated and disintegrated MCs, and less significant total number of MCs compared with the control group
Fridoni et al., 2019	Human bone marrow	Aspiration	Flow cytometry (CD105+, CD90+, CD73+, CD34−, CD45−)	MSCs of passage 4 were used. CM was collected	Murine. MRSA diabetic rats infected. Full−thickness wound, 15-mm diameter round, on the back	PBM was administered once daily, 6 days per week. 500 μl of the 10-fold CM was injected intraperitoneally daily from day 0 until day 3 - Control group - PBM - MSC-CM - PBM+MSC-CM (*n* = 18/group)	15	Histology (HE), IHC	PBM+MSC-CM hastened wound healing process	PBM+MSC-CM, MSC-CM, and PBM groups showed a decrease in the number of neutrophils and macrophages and an increase in the number of fibroblasts and angiogenesis compared with those of the control group
Aryan et al., 2019	Human bone marrow	−	Flow cytometry (CD73+, CD90+, CD105+, CD45−)	MSCs of passages 3–4 at 80–90% confluence were used. CM was collected	Murine. Second-degree burns (induced from boiling water), 30 mm × 30 mm	- Not treated rats (control) - 0.5 ml of DMEM injected intraperitoneally every other day - 1% topical silver sulfadiazine cream daily - 0.5 ml of MSC-CM injected intraperitoneally every other day (*n* = 5/group)	28	Macroscopic appearance (photography), histology (HE, MT), IHC	Wound closure area was significantly increased in the MSC-CM and sulfadiazine groups	There was a reduction in the volume of the epidermis and dermis in the burn wound of the control, DMEM, and sulfadiazine groups compared with the MSC-CM group

*CM, conditioned medium; DMEM, Dulbecco’s modified Eagle medium; ECM, extracellular matrix; EGF, epidermal growth factor; HE, hematoxylin and eosin; IHC, immunohistochemistry; IF, immunofluorescence; MRSA, methicillin-resistant *Staphylococcus aureus*; MSCs, mesenchymal stromal cells; MT, Masson’s trichrome; PBM, photobiomodulation; PBS, phosphate-buffered saline; qRT-PCR, real-time quantitative polymerase chain reaction.*

Twenty-eight mice with burn wounds were treated with MSC-CM with a mean follow-up of 25.67 days (from 21 to 28 days). MSC-CM was tested using subcutaneous (Li J. Y. et al., 2019) or intraperitoneal (Aryan et al., 2019) injections or topically applied (Zhou et al., 2019). In burn wounds, MSC-CM showed faster wound healing, increased re-epithelialization, and vascularization compared with controls (unconditioned medium, PBS, or DMEM).

Moreover, 72 mice infected with methicillin-resistant *Staphylococcus aureus* were treated with MSC-CM or MSC-CM plus photobiomodulation (PBM). MSC-CM plus PBM decreased colony-forming units and the number of inflammatory cells (Fridoni et al., 2019; Kouhkheil et al., 2019).

Twelve mice with radiation-induced skin injuries were also treated with MSC-CM. Topical application of MSC-CM in hydrogel accelerated wound closure and enhanced the wound healing quality (Sun et al., 2019).

##### Combined therapies using mesenchymal stromal cell–conditioned medium

Five studies evaluated the effects of MSC-CM combined with PBM or pulsed wave low-level laser therapy (PWLLLT) for treating diabetic wounds and infected wounds (Pouriran et al., 2016; Amini et al., 2018; Bagheri et al., 2018; Fridoni et al., 2019; Kouhkheil et al., 2019) (Supplementary Table 1). Regarding diabetic wounds, the results are controversial (Pouriran et al., 2016; Amini et al., 2018; Bagheri et al., 2018). Two studies showed that MSC-CM, PBM, and the combined therapy improved wound healing as compared with control group (Pouriran et al., 2016; Bagheri et al., 2018). Moreover, it was found that the extent of healing was significantly greater in the MSC-CM+PBM group (Amini et al., 2018). On the other hand, it was showed that PWLLLT and MSC-CM, alone or in combination, improved biomechanical parameters in the wound but that PWLLLT was more effective compared with MSC-CM (Pouriran et al., 2016). Concerning infected wounds, it was observed that both PBM+MSC-CM and PBM groups decreased colony-forming units and hastened wound healing process as compared with controls, while it did not happen when using MSC-CM alone (Fridoni et al., 2019; Kouhkheil et al., 2019).

#### Clinical Studies

Only one clinical study evaluated the effects of MSC-CM on wound healing (Table 4). This research assessed the use of MSC-CM derived from human amniotic membrane for treating chronic plantar ulcers in leprosy. The mean age of the patients was 52.12 ± 1.33 years, the mean ulcer duration was 1.41 ± 0.36 years, and the mean ulcer size at baseline was 2.64 ± 0.5 cm^2^ with a depth lower than 0.5 cm. Sixty-six patients were divided into groups to receive MSC-CM, MSC-CM+vitamin C, or MSC-CM+vitamin E topically applied every 3 days for 8 weeks. All groups improved wound healing, with MSC-CM+vitamin E being the most effective treatment. Wound size was reduced by 1.7 ± 1.05 vs. 2.01 ± 1.19 vs. 2.84 ± 1.67 cm^2^; and depth was decreased by 0.35 ± 0.14 vs. 0.25 ± 0.11 vs. 0.27 ± 0.15 cm in MSC-CM, MSC-CM+vitamin C, and MSC-CM+vitamin E groups, respectively. No adverse events were reported (Prakoeswa et al., 2018).

**TABLE 4:** Studies regarding mesenchymal stromal cell-conditioned medium for treating wounds in human.

	**MSC source**	**Method of tissue extraction**	**Indication**	**Study type**	**Age (years)**	**Sex (male:female)**	**Groups of treatments and via of administration**	**Follow-up (days)**	**Assessment**	**Main outcome**
Prakoeswa et al., 2018	Human amniotic fluid	Amniocentesis	Chronic plantar ulcer in leprosy	Randomized controlled trial	52.18 ± 1.33	5:6	Topical application every 3 days - MSC-CM - MSC-CM+vitamin C - MSC-CM+vitamin E (*n* = 22/group)	56	Macroscopic appearance (photography), spectrophotometric	All groups reduced wound size and depth. Healing percentage increased in all groups. Size reduction was 1.7 ± 1.05 vs. 2.01 ± 1.19 vs. 2.84 ± 1.67 cm^2^, and depth was decreased 0.35 ± 0.14 vs. 0.25 ± 0.11 vs. 0.27 ± 0.15 cm in MSC-CM, MSC-CM+vitamin C, and MSC-CM+vitamin E group, respectively. No adverse events were reported

*CM, conditioned medium; MSC, mesenchymal stromal cells.*

### Hypertrophic and Keloid Scars

#### Preclinical Studies

Nine studies evaluated the potential of MSC-CM in the treatment of hypertrophic scars or avoiding scar formation (Wu et al., 2015; Zhang et al., 2015; Du et al., 2016a, b; Li et al., 2016; Choi et al., 2017; Liu et al., 2018; Arjunan et al., 2020; Hu et al., 2020; Table 5). To obtain CM, MSCs were mainly isolated from human tissues: adipose tissue (Li et al., 2016; Choi et al., 2017; Liu et al., 2018) (33.33%, 3/9), umbilical cord blood (Arjunan et al., 2020), placenta (Du et al., 2016a), and bone marrow (Wu et al., 2015). Murine bone marrow (Hu et al., 2020), murine placenta (Du et al., 2016b), and rabbit adipose tissue (Zhang et al., 2015) were also used. CM was collected from MSCs between passages 3 and 13 at 70–90% confluence. The most common model was murine (88.89%, 8/9), although a rabbit model was also used (Zhang et al., 2015). CM was used mainly by subcutaneous injection (66.66%, 6/9) (Wu et al., 2015; Du et al., 2016a, b; Li et al., 2016; Liu et al., 2018; Hu et al., 2020). Intralesional (Zhang et al., 2015; Arjunan et al., 2020) and intravenous (Choi et al., 2017) injections were also used. MSC-CM was tested in 97 animals for treating hypertrophic scar and keloids or preventing scar formation. The mean follow-up time was 23 days. Scars were assessed mainly by macroscopic appearance, histology, immunohistochemistry, and qRT-PCR.

**TABLE 5:** Studies regarding mesenchymal stromal cell-conditioned medium for treating hypertrophic scars in animal models.

	**MSC source**	**Method of tissue extraction**	**MSC characterization**	**MSC treatment**	**Model**	**Groups of treatments and via of administration**	**Follow-up (days)**	**Assessment**	**Main outcome**	**Other outcomes**
Arjunan et al., 2020	Human umbilical cords	Dissection	Flow cytometry and IF (Tra-1–60, Tra-1–81, SSEA-1, SSEA-4, Oct-4 and alkaline phosphatase, CD105, CD90, CD44)	MSCs at 70–89% confluence were used. CM was then collected	Murine. Keloid xenograft SCID mouse model, 3-6 mm, limbs	50 μl intralesional injected - Placebo - HSF-CM - MSC- CM (*n* = 9/group)	30	Macroscopic appearance	MSC-CM group showed greater keloid reduction	A reduction in keloid tumor volumes (12.04 ± 3.69 vs. 54.65 ± 8.97 vs. 71.78 ± 20.67 mm) and weights (26.50 ± 6.38 vs. 76.70 ± 9.58 vs. 73.70 ± 12.12 mg) in the WJ-MSC-CM group compared with HSF-CM and untreated group were observed
Hu et al., 2020	Murine bone marrow	Needle aspiration from tibia and femur	Flow cytometry (CD150+/CD74+). Osteogenic, adipogenic, chondrogenic differentiation	MSCs of passages 8–13 at 70% confluence. CM was then collected	Murine. Human HS-buried null mouse model, 6 mm, back	200 μl subcutaneously injected every 7 days - DMEM - Botox - MSC-CM - MSC-CM+Botox (*n* = 4/group)	28	Macroscopic appearance, histology, IF, collagen deposition assay, fibroblast apoptosis assay (caspase-7 staining), qRT-PCR, WB	HS was the most reduced in MSC-CM+Botox group compared with the other groups. Scar weight was reduced up to 70% in MSC-CM+Botox, up to 80% in MSC-CM, up to 81% in botox, and up to 91% in control group	Collagen fiber deposition was eased and well-arranged after all treatments, except control group. α-SMA expression was lower in the combined regimen, MSC-CM, and botox than in DMEM group
Liu et al., 2018	Human subcutaneous adipose tissue	Surgical excision of redundant tissue from surgical operations	Flow cytometry (CD105+/CD90+/ CD34−/CD45−/ CD19−). Adipogenic and osteogenic differentiation	MSCs of passages 3–4 at 80% confluence were used. CM was then collected	Murine. Keloid xenograft, 10 mm, back	200 μl injected subcutaneously into each keloid xenograft every week - Untreated - DMEM - MSC-CM (*n* = 4/group)	28	Macroscopic appearance, histology, IHC, BrdU proliferation assay (bromodeoxyuridine/5-bromo-2′-deoxyuridine) (ELISA), qRT-PCR, contraction assay, phosphatidylserine apoptosis assay, antibody-based array	MSC-CM had a greater effect in scar reduction than control group. MSC-CM group decreased HS weight, by 34% compared with untreated group and by 23% compared with DMEM group	MSC-CM reduced the proportion of both cellularity/inflammatory cells and blood vessel density
Choi et al., 2017	Human subcutaneous adipose tissue	Cesarean section	−	MSCs of passage 3 at 70–80% confluence were used. CM was then collected, and exosomes were created	Murine. Full-thickness wound scar, 20 mm × 15 mm, on dorsal surface	200 μl intravenously injected - PBS - Exos-free CM - Exos-MSCs (*n* = 6/group)	21	Macroscopic appearance (photography), histology (HE, MT, picrosirius red), IHC, qRT-PCR	MSC-Exos treatment attenuated the thickness of the dermal layer and the length of the scar	The surface of the epidermis was more flattened, and collagen in the dermis was well distributed with less crosslinking in MSC-Exos group. MSC-Exos reduced collagen deposition, mitigated scar formation, and increased the ratio of collagen III
Li et al., 2016	Human adipose tissue	Liposuction	Flow cytometry (CD73+/CD90+/ CD34−/CD14−), adipogenic and osteogenic differentiation	MSCs of passages 3–5 at 80–90% confluence were used. CM was then collected	Murine. Full-thickness excisional wound, 10 mm, back	1,000 μl subcutaneously injected into each scar at four points - DMEM - MSC-CM (*n* = 6/group)	14	Macroscopic appearance (photography), histology (HE and Masson trichrome staining), immunohistochemistry, qRT-PCR, WB	Reduced scar formation and fibrosis were observed in MSC-CM group	MSC-CM decreased the expression of Col1, Col3, and α-SMA
Du et al., 2016a	Murine placenta	Placenta collected	Flow cytometry (CD29+, CD31−, CD34−, CD44+, CD45− and HLA-DR−)	MSCs of passage 3 were used. Normoxic CM, hypoxic CM, and hypoxic plus inhibitor CM (HIF-1α) were collected	Murine. Hypertrophic scald mouse model, 20 mm, back	100 μl injected subcutaneously - Control - Normoxic MSC-CM - Hypoxic MSC-CM - HIF-1a inhibitor+hypoxic MSC-CM (*n* = 10/group)	15	Macroscopic appearance, histology (HE), WB	MSC-CM reduced scar formation	MSC-CM attenuated inflammatory responses and decreased the deposition of collagens
Du et al., 2016b	Human placenta	Placenta collected	Flow cytometry and immunofluorescence positive for CD73+, CD90+, CD105+, CD34−, CD45−, HLA-DR−	MSCs of passages 3–6 at 80–90% confluence were used. Normoxic CM and hypoxic CM were collected	Murine. Hypertrophic scald mouse model, 20 mm, back	100 μl injected subcutaneously - Normal medium - Normoxic MSC-CM - Hypoxic MSC-CM (*n* = 10/group)	15	Macroscopic appearance (photography), contracture rate histology (HE), Trypan blue staining, qRT-PCR, WB, ELISA, wound healing assay	Hypoxic MSC-CM reduced scar formation and decreased wound size compared with normal medium and normoxic MSC-CM	Decreased levels of TGF-β1 and collagen I were observed using hypoxic MSC-CM. Hypoxic MSC-CM also inhibited the proliferation and migration of skin fibroblasts
Wu et al., 2015	Human bone marrow	−	Flow cytometry (CD14, CD34, CD45, CD44, and CD73)	MSCs of passages 5–10 at 80% confluence were used. CM was then collected	Murine. Skin fibrosis model. Hypertrophic skin, almost 2-fold greater thickness than normal skin, back.	100 μl injected subcutaneously into the lesion every - Placebo - MSC-CM - BM-MSC-CM with TGF-β3 blocked (*n* = 5/group)	21	Macroscopic appearance, histology (HE), IHC, cellular proliferation (Ki-67)	MSC-CM decreased skin fibrosis	MSC-CM decreased the fibroblast viability, reduced skin dermal thickness, and inhibited cells proliferation. Moreover, MSC-CM-treated group exhibited significantly fewer proliferating cells compared with MSC-CM with TGF-β3-blocked
Zhang et al., 2015	Rabbit adipose tissue	Surgical excision of inguinal fat pad	Flow cytometry (CD73+/CD90+/ CD34−/CD14−). Adipogenic and osteogenic differentiation	MSCs of passage 3 at 80–90% confluence were used. CM was then collected	Rabbit. Full-thickness wound scar, 10 mm, ear	200 μl intralesionally injected into center of each scar - Untreated (*n* = 4) - MSCs (*n* = 4) - MSC-CM (*n* = 4) - DMEM (*n* = 12)	35	Macroscopic appearance, histology (HE, MT), IHC, qRT-PCR, gene expression, ultrasonography	MSCs and MSC-CMs decreased scar hypertrophy and led to scar with a more normal appearance	MSCs and MSC-CM decreased α-smooth muscle actin and collagen type I. MSC-CM was less effective than MSCs

*BM, bone marrow; CM, conditioned medium; DMEM, Dulbecco’s modified Eagle medium; ELISA, enzyme-linked immunosorbent assay; Exos, exosomes; HE, hematoxylin and eosin; HS, hypertrophic scar; HSF, human skin fibroblast; IF, immunofluorescence; IHC, immunohistochemistry; MSCs, mesenchymal stromal cells; MT, Masson’s trichrome; qRT-PCR, real-time quantitative polymerase chain reaction; WB, Western blotting; WJ, Wharton’s jelly.*

Mesenchymal stromal cell-CM had better results than non-treated groups or other treatments groups (PBS, DMEM, and unconditioned medium) in terms of tumor volumes, tumor weight, collagen fiber deposition, and skin fibrosis. CM derived from MSCs cultured in hypoxic conditions showed greater effects on scar formation than cultured in normoxic conditions (Du et al., 2016a, b). Moreover, the MSC-CM-treated group exhibited significantly fewer proliferating cells than did MSC-CM with TGF-β3 blocked, showing that TGF-β3 may be responsible for parts of the antifibrotic effect of the MSC-CM (Wu et al., 2015). The combination of MSC-CM and botulinum toxin type A (botox) had a greater effect when treating human hypertrophic scars than did botox alone, MSC-CM alone, or DMEM. Scar weight was reduced by 21, 11, or 10% more compared with DMEM, MSC-CM, or botox, respectively (Hu et al., 2020). Although both MSCs and MSC-CMs reduced hypertrophic scars, MSC-CM might be more effective than MSCs (Zhang et al., 2015). The Scar Elevation Index was reduced in both MSC- and MSC-CM-treated groups compared with internal controls (44.04 and 32.48%, *p* < 0.01, respectively), and it was significantly lower in the MSC-treated group than in the MSC-CM-treated group (*p* < 0.01) (Zhang et al., 2015).

#### Clinical Studies

No clinical study specifically evaluated the effects of MSC-CM on hypertrophic scar formation. Nevertheless, the effect of MSC-CM has been tested on acne scars in combination with laser therapy, showing that combined therapy had a greater effect on reducing acne scars than laser alone (Zhou et al., 2016; Abdel-Maguid et al., 2019; El-Domyati et al., 2019; Park C. S. et al., 2019). These studies are further developed in the Skin Rejuvenation section.

### Flap and Graft Reperfusion

#### Preclinical Studies

Four studies evaluated the effects of MSC-CM for treating ischemic wounds or flaps (Mirabella et al., 2012; Lee S. M. et al., 2014; Cooper et al., 2018; Pu et al., 2019; Table 6). MSCs were isolated from human adipose tissue (Lee S. M. et al., 2014; Cooper et al., 2018; Pu et al., 2019) or human amniotic fluid (Mirabella et al., 2012). CM was collected from MSCs between passages 3 and 8 at 70–90% confluence. All the models employed were murine.

**TABLE 6:** Studies regarding mesenchymal stromal cell-conditioned medium for improving flaps reperfusion in animal models.

	**MSC source**	**Method of tissue extraction**	**MSC characterization**	**MSC treatment**	**Model**	**Groups of treatments and via of administration**	**Follow-up (days)**	**Assessment**	**Main outcome**
Pu et al., 2019	Human abdominal adipose tissue	Liposuction	Flow cytometry and IHC (CD34, CD45, CD73, CD90, CD105). Adipogenic, osteogenic, chondrogenic differentiation	MSCs of passages 3–8 at 70–80% confluence were used. CM was then collected	Murine. Ischemia/reperfusion flap model by clamping the flap (ischemia) and then releasing it (reperfusion)	Treatment was applied to the subcutaneous layer between the flap and its bed and at the proximal, middle, and distal parts of the skin flap - Saline injections - MSC-CM (*n* = 6/group)	5	Macroscopic appearance (photography), histology (HE, MT), IHC, qRT-PCR	MSC-CM treatment attenuated the necrotic area and reversed the detrimental proliferation effect induced by I/R compared with saline
Cooper et al., 2018	Human adipose tissue	Elective surgery	−	MSCs of at 70–90% confluence were used. CM was then collected. EVs were isolated by centrifugation	Murine. Full-thickness excisional wounds were created in the center of a 105 mm × 35 mm flap (ischemic wounds)	20 μl topically applied daily - MSC-CM - Control media (*n* = 6/group)	21	Macroscopic appearance (photography)	MSC-CM accelerated closure of ischemic wounds. In MSC-CM-treated group, the wound area reduction was 65% compared with only 15% wound closure for the control
Lee S. M. et al., 2014	Human subcutaneous adipose tissue	−	Flow cytometry (CD29+, CD44+, CD73+, CD90+, CD105+, CD34−, CD45−, CD14−)	MSCs of passage 3 at 80% confluence were used	Murine. Full-thickness skin graft, 30 mm × 30 mm, back	Intravenously injected - PBS - MSCs - MSC-CM (*n* = 12/group)	30	Macroscopic appearance (photography), histology (HE), IHC	MSCs and MSC-CM increased skin allograft survival compared with control (23.9 ± 2.0, 19.6 ± 2.4, and 9.3 ± 1.4 days, respectively), without differences between these both groups
Mirabella et al., 2012	Human amniotic fluid	Amniocentesis	−	MSCs of passage 6 at 80% confluence. Lyophilized gelatine film membranes were prepared by using MSC-CM or saline as control	Murine. Ischemia/reperfusion epigastric flap model. Flap 70 mm × 70 mm with vasculature ligated and then opened	A membrane with MSC-CM or saline was positioned under the elevated skin flap (*n* = 5/group)	7	Macroscopic appearance (photography), histology	Perfusion was increased in flaps treated with MSC-CM compared with controls. Necrosis was decreased in the MSC-CM group

*CM, conditioned medium; EVs, extracellular vesicles; HE, hematoxylin and eosin; IHC, immunohistochemistry; I/R, ischemia/reperfusion; IF, immunofluorescence; MSCs, mesenchymal stromal cells; MT, Masson’s trichrome; PBS, phosphate-buffered saline; qRT-PCR, real-time quantitative polymerase chain reaction.*

Two studies tested MSC-CM in ischemia/reperfusion flap models by clamping the flap and then releasing it. Pu et al. evaluated the effects of MSC-CM applied to the subcutaneous layer between the flap and its bed and at the proximal, middle, and distal parts as compared with saline (*n* = 6/group) (Pu et al., 2019). A porcine skin-derived gelatine membrane soaked in either MSC-CM or saline was also tested (*n* = 5/group) (Mirabella et al., 2012). Both studies observed that the MSC-CM treatment group enhanced skin flap recovery, attenuated the necrotic area, increased hair growth, and stimulated angiogenesis as compared with controls (Mirabella et al., 2012; Pu et al., 2019).

MSC-CM were also applied topically on flap ischemic wounds in six mice. It was observed that MSC-CM accelerated the closure of flap ischemic wounds by 50% as compared with controls (Cooper et al., 2018). MSC-CM was also tested on skin allografts, intravenously injected, and compared with placebo and MSCs. MSCs and MSC-CMs increased skin allograft survival as compared with the control (23.9 ± 2.0, 19.6 ± 2.4, and 9.3 ± 1.4 days, respectively), without any differences between MSCs and MSC-CMs (Lee S. M. et al., 2014).

There are no studies at the clinical level.

### Hair Restoration

#### Preclinical Studies

Ten studies evaluated the effect of MSC-CM on hair growth at the preclinical level (Park et al., 2010; Won et al., 2010, 2015; Dong et al., 2014; Shim, 2015; Choi et al., 2019; Gunawardena et al., 2019a; Park J. et al., 2019; Ou et al., 2020; Xiao et al., 2020; Table 7). MSCs were isolated mainly from human hair follicles (Won et al., 2010; Won et al., 2015; Gunawardena et al., 2019a; Ou et al., 2020) (40%, 4/10) and human adipose tissue (Park et al., 2010; Choi et al., 2019; Xiao et al., 2020) (30%, 3/10). Other tissues used were human amniotic fluid (Park J. et al., 2019), human deciduous teeth (Gunawardena et al., 2019a), human dermal cells (Shim, 2015), and murine bone marrow (Dong et al., 2014). CM was collected from MSCs between passages 2 and 6 at 50–100% confluence. All studies employed murine models.

**TABLE 7:** Studies regarding mesenchymal stromal cell-conditioned medium for hair restoration in animal models.

	**MSC source**	**Method of tissue extraction**	**MSC characterization**	**MSC treatment**	**Model**	**Groups of treatments and via of administration**	**Follow-up (days)**	**Assessment**	**Main outcome**	**Other outcomes**
Xiao et al., 2020	Human adipose tissue	Fat grafting	IF (alkaline phosphatase+ and β-catenin+)	MSCs of passage 3 were used. CM from MSCs and from ECM/SVF-gel was collected	Murine. Back hair shaved with clippers and then completely removed using hair remover cream	Injected subcutaneously in the dorsal skin once per week - PBS (control) - ECM/ADSVC-CM - ADSVC-CM (*n* = 10/group)	21	Macroscopic appearance (skin darkening, hair growth score, gross observation of the inner skin of the hair-regrowth), IHC (CD31), WB	Both CMs enhanced hair growth. CM from ECM/SVF had a stronger ability to stimulate hair growth. 95–100% hair regeneration to full length was observed in the ECM/SVF group and 70–75% in the ADSVC-CM group	Both CM promoted dermal papilla cells and bulge cell proliferation, neovascularization, and anagen induction. Growth factor levels (VEGF, bFGF, PDGF, KGF) increased in CM-treated mice, even more than in ECM/ADSVC-CM group
Ou et al., 2020	Donor hair follicles from the adult occipital scalp	Biopsy sample of hair follicles	−	MSCs at 80% confluence were used. CM was then collected	Murine. Left and right body of each rat shaved as close as possible to the skin	Gel topically applied - Vehicle (control) - MSC-CM (*n* = 4/group)	15	Macroscopic appearance (photography), histology (HE)	No significant differences in hair growth were observed between MSC-CM and control group	MSC-CM-treated group showed a darker area of the skin and bigger diffusion. Histologically, the hair follicle cycling was enlarged in the MSC-CM-treated group
Park J. et al., 2019	Human amniotic fluid	Amniocentesis performed for fetal karyotyping between 16- and 20-week gestation	IF and quantification (CD13+, CD29+, and CD44+). Osteogenic, adipogenic, and chondrogenic differentiation	MSCs of passage 3 at 70–80% confluence were used. CM was then collected	Murine. Plucking the dorsal skin of mice in the telogen phase of the hair cycle, inducing the second anagen stage	50 μl topically applied - MSC-CM - MSC-Nanog-CM - Minoxidil 2% (control) (*n* = 3/group)	10	Macroscopic appearance (photography), histology (HE), IHC (ALP, CK15), qRT-PCR, WB (ALP, LEF1, and Versican)	The dorsal skin of MSC-Nanog-CM and minoxidil-treated mice was fully darkened, while bare spots were observed in MSC-CM group	MSC-Nanog-CM accelerated the telogen-to-anagen transition in HFs and increased HF density to a greater extent than MSC-CM. The expression of DP and HF stem cell markers and genes related to hair induction were higher in MSC-Nanog-CM than in AF-MSC-CM. Paracrine factors contained in MSC-Nanog-CM
										upregulated the expression of hair induction genes and accelerated hair regeneration
Gunawardena et al., 2019	hDP-MSCs: human deciduous teeth hHF-MSCs: human donor hair follicles	hDP-MSCs: teeth extraction hHF-MSCs: scalp biopsy	Flow cytometry (CD73+, CD90+, CD105+). Adipogenic, chondrogenic, osteogenic differentiation	SHED of passages 2–5 and HFSCs of passage 1 at 80% confluence were used. CM was then collected	Murine. Dorsal region shaved with clippers	100 μl subcutaneously injected at the dorsal region every 3 days - hDP-MSCs -CM (*n* = 9) - hHF-MSC-CM (*n* = 9) - STK2 media, control (*n* = 3) - No treatment, control (*n* = 2)	60	Macroscopic appearance (photography)	hDP-MSC-CM-treated group showed a faster stimulation of hair growth in comparison with hHF-MSC-CM. The visual observance for the appearance of dark patches indicating the transition from telogen to anagen stage by hDP-MSC-CM ranged from 8 to 12 days while hHF-MSC-CM ranged from 12 to 15 days	VEGF-A and HGF were overexpressed in hDP-MSC-CM and hHF-MSC-CM group. No adverse events were reported
Choi et al., 2019	Human adipose subcutaneous fat	Liposuction	−	MSCs were of passages 4–6 were used. Cells were seeded and treated with HB-EGF. CM was then collected.	Murine. Dorsal region shaved with clippers	Subcutaneously injected - ADSC-CM - ADSC-HB-EGF- CM (*n* = 4/group)	17	Macroscopic appearance (photography), histology (HE), IHC, qRT-PCR	MSC-CM and MSC-HB-EGF-CM promoted hair growth. MSC-HB-EGF-CM showed higher increase in hair weight	MSC-HB-EGF-CM more rapidly induced telogen-to-anagen hair cycling and showed higher number of mature hair follicles. Expression levels of several growth factors in the MSC-HB-EGF-CM were upregulated compared with MSC-CM group
Won et al., 2015	Donor hair follicles	CELLnTEC	Flow cytometry (integrin a6 and CD71)	MSCs were obtained from CELLnTEC. MSCs were used	Murine. Dorsal side shaved with a clipper and electric shaver	Subcutaneously injected every 2 days - PBS (control)	14	WB	MSC-CM-treated group increased hair growth compared	MSC-CM enhanced the proliferation of HFs. The level of
				at 100% confluence. CM was then collected		- MSC-CM (*n* = 5/group)			with controls	phosphorylated AKT and phosphorylated ERK1/2 was significantly increased after MSC-CM treatment
Shim, 2015	Human dermal cells	−	Flow cytometry. Adipogenic, chondrogenic, osteogenic differentiation	MSCs of passage 2 were used. CM was then collected	Murine. Dorsal region shaved with a clipper	100 μl topically applied daily - MSC-CM or - Minoxidil - Non-treated group (*n* = 6/group)	35	Visual scoring of hair growth	MSC-CM increased hair regeneration. After the follow-up, hair weight was around 20, 40, and 80 mg in control, minoxidil, and MSC-CM group, respectively	MSC-CM increased promoted early telogen-to-anagen phase conversion of hair follicles compared with minoxidil treated group and non-treated mice
Dong et al., 2014	Murine bone marrow from the femur	Needle aspiration	Flow cytometry (CD29+, CD44+, CD73+, CD14−, CD34, CD45, CD133−). Adipogenic, chondrogenic, osteogenic differentiation	MSCs were transfected with murine Wnt1a cDNA retroviral vector. MSCs and Wnt1a-MSCs at 50–60% confluence were used. CM was collected from MSCs and Wnt1a-MSC-	Murine. Dorsal region shaved with a clipper	100 ml intradermally injected - Wnt-CM - MSC-CM - DMEM (control) (*n* = 3/group)	14	Macroscopic appearance (photography), histology (HE), IHC, qRT-PCR	Both Wnt-CM and MSC-CM promoted the hair follicle cycling more than control group	Wnt-CM induces hair to enter earlier anagen of the hair cycle. ALP expression was enhanced in both MSC-CM and Wnt-CM group. More Ki67-positive cells were observed in Wnt-CM-treated mice. Both MSC-CM and Wnt-CM upregulated the hair induction-related genes
Won et al., 2010	Human hair follicles	Dissection	IHC (alpha smooth muscle actin, alkaline phosphatase)	MSCs were transfected with plasmid carrying SV40T antigen and c-myc gene and neomycin-resistant gene. MSCs of passages 3–4 were used. CM was collected	Murine	Topical application - MSC-CM - DMEM (*n* = 3/group)	21	Macroscopic appearance (photography)	MSC-CM enhanced hair growth. Hair weight was 2- to 5-fold higher in MSC-CM compared with control group	MSC-CM accelerated hair growth
Park et al., 2010	Human subcutaneous adipose tissue	Liposuction	−	MSCs of passages 4–5 were used. MSCs were cultured in nomoxic and hypoxic conditions. CM was then collected	Murine	Subcutaneously injected - Normoxic MSC-CM (*n* = 5) - Hypoxic MSC-CM (*n* = 5) - Control (*n* = 3)	56	Macroscopic appearance (photography)	MSC-CM increased hair regeneration compared with control	MSC-CM induced the anagen phase. Hair regeneration growth factors significantly increased by hypoxia

*ADSCs, adipose-derived stem cells; ADSVCs, adipose-derived stromal vascular cells; BM, bone marrow; DP, dermal papilla; hDPs, human dermal papilla cells; ECM, extracellular matrix; HF, hair follicle; hFDPs, human follicle dermal papilla cells; hHF-MSCs, human hair follicle mesenchymal stromal cells; hDSPC, human dermal stem/progenitor cell; hDP-MSC, human dental pulp mesenchymal stromal cells; IF, immunofluorescence IHC, immunohistochemistry; KSCs, keratinocyte stem/progenitor cells; MSCs, mesenchymal stromal cells, PBS, phosphate-buffered saline; SHED, dental pulp stem cells obtained from human deciduous teeth; SVF, stromal vascular fraction; WB, Western blotting.*

Mesenchymal stromal cell-CM was used subcutaneously (Park et al., 2010; Won et al., 2015; Choi et al., 2019; Gunawardena et al., 2019a; Xiao et al., 2020) (50%, 5/10) or intradermally (Dong et al., 2014) injected or topically applied (Won et al., 2010; Shim, 2015; Park J. et al., 2019; Ou et al., 2020) (40%, 4/10). MSC-CM was tested on 86 animals with shaved regions. The mean follow-up time was 26.3 days (from 10 to 60 days). Hair growth improvement was assessed mainly by macroscopic appearance (skin darkening and hair weight) or histology. It was found that MSCs enhanced hair growth compared with control group (PBS, minoxidil, or DMEM). Hair weight in the MSC-CM group increased by 40 mg as compared with that in the non-treated groups and by 20 mg as compared with that in the minoxidil group (Shim, 2015). MSC-CM stimulated hair growth by promoting dermal papilla cell proliferation, accelerating telogen-to-anagen transition, and promoting neovascularization.

The overexpression of a reprogramming factor in the CM, *Nanog*, improved hair induction even more than MSC-CM without Nanog (Park J. et al., 2019). Moreover, it was observed that CM collected from human deciduous teeth accelerated hair growth more than CM derived from hair follicle cells. The transition from telogen to anagen stage for teeth-MSC-CM was between 8 and 12 days, while that at hair follicle-MSC-CM ranged from 12 to 15 days (Gunawardena et al., 2019a). CM enrichment with heparin binding-epidermal growth factor-like growth factor (HB-EGF) showed a greater effect in promoting hair growth compared with non-supplemented CM (Choi et al., 2019). Furthermore, CM collected from MSCs cultured in hypoxic conditions increased hair regeneration growth factors (Park et al., 2010). No adverse events were reported when using MSC-CM in hair restoration.

#### Clinical Studies

Four clinical studies evaluated the effects of MSC-CM on hair growth (Shin et al., 2015; Lee et al., 2020; Narita et al., 2020; Oh et al., 2020; Table 8). To obtain CM, cells were isolated from human adipose tissue (Shin et al., 2015; Lee et al., 2020; Narita et al., 2020) (75%) and human umbilical cord blood (Oh et al., 2020) (25%). All studies evaluated the effect of MSC-CM in androgenetic alopecia (AGA). MSC-CM was tested on 100 patients, both men and women, with an age range of 23–74 years. Two observational studies used intradermal injections of human AT-MSC-CM and observed that hair density increased by 17.3 hairs/cm^2^ and hair thickness increased by 6.5 μm as compared with baseline (Shin et al., 2015; Narita et al., 2020). A clinical trial also evaluated the effects of human UC-MSC-CM enriched with TGF-1 and LiCl applied topically twice daily for 112 days. It was observed that MSC-CM increased hair density by 14.24%, hair thickness by 28.19%, and hair growth by 19.54% as compared with a placebo (Oh et al., 2020). Moreover, MSC-CM treatment was also evaluated in patients who underwent non-ablative fractional laser treatment for AGA. It was found that concomitant treatment (MSC-CM+laser) had a greater effect than laser treatment only. The MSC-CM group had greater hair density (102.1 ± 4.09 vs. 89.3 ± 3.79/cm^2^) and gross hair volume (2 ± 0.13 vs. 1.2 ± 0.19, evaluated by a 7-point global improvement score) than the placebo group after 12 weeks of treatment administration (Lee et al., 2020). No serious adverse events were reported in these studies.

**TABLE 8:** Studies regarding mesenchymal stromal cell-conditioned medium for hair restoration in humans.

	**MSC source**	**Method of tissue extraction**	**MSC characterization**	**MSC treatment**	**Indication**	**Study type**	**Age (years)**	**Sex (male: female)**	**Groups of treatments and via of administration**	**Follow-up (days)**	**Assessment**	**Main outcomes**
Oh et al., 2020	Human umbilical cord	Umbilical cord section	Flow cytometry (CD14, CD45, HLA-DR, PE-conjugated human CD73, CD166, BD, CD90, and CD105)	MSCs of passaged 5 at 60–70% confluence were used. CM enriched with TGF-1 and LiCl (primed-MSC-CM). CM was collected without pretreatment with TGF-1 and LiCl to act as the control	Healthy adults diagnosed with AGA (males: Type II according to the modified Norwood–Hamilton classification, women: Ludwig classification Type I)	Double blind placebo-controlled clinical trial	46.9 (range 33–55)	1:30	5% CM topically applied twice daily - Primed-MSC-CM (*n* = 16) - Placebo (*n* = 14)	112	Hair density and diameter (phototrichogram), hair density (hair count/cm^2^, counting the total number of hairs in the target area), hair thickness (mm) and hair growth rate (mm/day), rate of hair growth	Primed-MSC-CM improved androgenetic alopecia. Hair density increased by 14.24% in primed-MSC-CM group, while no improvement was observed in placebo group. Hair thickness increased by 28.19%, and hair growth rate increased by 19.54% in primed-MSC-CM-treated group. Primed-CM significantly increased the viability of DPCs
Narita et al., 2020	Human subcutaneous adipose tissues	Liposuction	Flow cytometry (CD73+/CD90+/ CD34−/CD14−). Adipogenic and osteogenic differentiation	MSCs of passage 4 were used. Cells were cultured under hypoxia conditions. CM was then collected	Patients with AGA	Prospective observational study	Range 23–74	21:19	Intradermal injection of ADSC-CM every month (*n* = 40)	180	Trichograms, physiological examinations (TEWL, SCH lipid level), ultrasonogray (dermal thickness and echogenicity), histology (HE, Elastica Masson–Goldner staining, Sirius red/fast green staining)	Hair density and anagen hair rate increased significantly compared with baseline. TEWL increased, while SCH and lipid level showed no obvious changes. Dermal thickness and dermal echogenicity increased significantly
Lee et al., 2020	Human subcutaneous adipose tissues	Liposuction	Flow cytometry (CD73+/CD90+/ CD34−/CD14−). Adipogenic and osteogenic differentiation	MSCs are cultured with hydrogel, and the CM is collected, after being filtered using 0.22-mm filters. Then, it is added to a solution, and SCM2-Black3 is obtained	Patients with AGA	Double-blinded, randomized placebo-controlled study	46.6 (range 20–61)	1:1	Before CM application, a single session of treatment was performed at the first visit and weekly single-pass self-applications of microneedle stamps. Topically applied once per week - MSC-CM - Normal saline (placebo) (*n* = 30)	84	Macroscopic appearance (photography), phototrichograms (hair density), gross hair volume improvement, investigator’s improvement (measured by questionnaire response)	MSC-CM group had significantly higher hair density than placebo (102.1 ± 4.09 vs. 89.3 ± 3.79/cm^2^). The gross hair volume of the MSC-CM group was also significantly higher (2 ± 0.13 vs. 1.2 ± 0.19/cm^2^). Investigator’s improvement was similar in both groups. No adverse effects associated with ADSC-CM were reported
Shin et al., 2015	Human subcutaneous adipose tissues	Liposuction	Flow cytometry (CD73+/CD90+/ CD34−/CD14−), Adipogenic and osteogenic differentiation	MSCs of passage 4 were used. Cells were cultured under hypoxia conditions. CM was then collected	Patients with AGA, female pattern hair loss	Retrospective observational study.	41.9 ± 13.4 (range 22–69)	0:27	Intradermal injection of MSC-CM every week (*n* = 27)	84	Patients’ medical records and phototrichographic images (hair density and thickness)	Hair density increased from 17.3 hairs/cm^2^, and hair thickness increased by 6.5 μm compared with baseline. None of the patients reported severe adverse events. The only inconvenience reported was the pricking of the needles during application

*AGA, androgenetic alopecia; CM, conditioned medium; DPC, dermal papilla cells; HE, hematoxylin and eosin; IHC, immunohistochemistry; IF, immunofluorescence; MSCs, mesenchymal stromal cells; MT, Masson’s trichrome; PBS, phosphate-buffered saline; qRT-PCR, real-time quantitative polymerase chain reaction; SCH, stratum corneum hydration; TEWL, transepidermal water loss.*

### Skin Rejuvenation

#### Preclinical Studies

Three studies evaluated the effect of MSC-CM in 36 UV-induced photoaged mice (Ueda and Nishino, 2010; Kwon et al., 2016; Zhang K. et al., 2020; Table 9). MSCs were isolated from human umbilical cord (33.33%, 1/3) (Zhang K. et al., 2020), human bone marrow (33.33%, 1/3) (Kwon et al., 2016), and human dental pulp (33.33%, 1/3) (Ueda and Nishino, 2010). The mean follow-up was 35 days (from 14 to 35). MSC-CM reduced the level of wrinkles, improved transepidermal water loss (TEWL) and stratum corneum hydration (SCH) values, and increased collagen fibers than did vehicle or PBS. Nevertheless, the effect of MSC-CM on improving wrinkles was lower than that of MSCs (Ueda and Nishino, 2010).

**TABLE 9:** Studies regarding mesenchymal stromal cell-conditioned medium for skin rejuvenation in animal models.

	**MSC source**	**Method of tissue extraction**	**MSC characterization**	**MSC treatment**	**Model**	**Groups of treatments and via of administration**	**Follow-up (days)**	**Assessment**	**Main outcomes**
Zhang K. et al., 2020	Human umbilical cord	Umbilical cord dissection	Western blotting (CD9+, CD63+, CD81+, and calnexin−)	MSC-CMs of passage 6 at 80–90% confluence were used. CM was collected, and Exos were obtained	Murine. Photoaged mouse model	- Control (non-photoaged mouse) - SHS-PBS - Exos - SHS-Exos (*n* = 6/group)	14	Macroscopic appearance, microscopic appearance (microwrinkles analysis), histology, and qRT-PCR	SHS-Exos reduced microwrinkles, alleviated histopathological changes, and promoted the expression of extracellular matrix constituents, while Exos alone produced weaker effects
Kwon et al., 2016	Human bone marrow from the posterior iliac crest	Aspiration	Flow cytometry (CD73+, CD105+, CD14−, CD34−, CD45−)	MSCs of passage 6 at 80% confluence were used and cultured in hypoxic conditions. CM was then collected	Murine. UVB-irradiated mice model	200 μl of treatment topically applied three times a week - Vehicle solution (polyethylene glycol:ethanol) - Adenosine 0.04% - MSC-CM 1% - MSC-CM 10% (*n* = 8/group)	56	Macroscopic appearance (photography, visual skin grading), tape stripping, biophysical measurements (TEWL and hydration), histology (HE, IHC)	The MSC-CM group exhibited significantly reduced levels of total wrinkle area compared with the vehicle-treated group. TEWL levels decreased while hydration increased in MSC-CM
Ueda and Nishino, 2010	Human dental pulp tissue	Healthy permanent deciduous teeth extraction	−	MSCs of passage 1 to 3 were used, and CM was collected	Murine. Wrinkled mice induced by UVB	Subcutaneous injections - MSC-CM - MSCs - PBS (*n* = 8/group)	35	Macroscopic appearance (photography, histology (HE)	MSCs and MSC-CM decreased wrinkles more than the PBS group. The greatest effect was observed in MSC group. Measurement of the dermal thickness showed significant increases in MSC and MSC-CM groups

*CM, conditioned medium; Exos, exosomes; HE, hematoxylin and eosin; IHC, immunohistochemistry; MSCs, mesenchymal stromal cells; MT, Masson’s trichrome; PBS, phosphate-buffered saline; qRT-PCR, real-time quantitative polymerase chain reaction; SHSs, sponge *Haliclona* sp. spicules; TEWL, transepidermal water loss.*

#### Clinical Studies

Twelve studies evaluated the effect of MSC-CM on skin wrinkles (Seo et al., 2013; Zhou et al., 2013; Lee S. M. et al., 2014; Xu et al., 2016; Kim et al., 2018; Prakoeswa et al., 2019; El-Domyati et al., 2020; Kim J. et al., 2020) and acne scars (Zhou et al., 2016; Abdel-Maguid et al., 2019; El-Domyati et al., 2019; Park C. S. et al., 2019; Table 10). MSCs were isolated from human tissues: adipose tissue (Zhou et al., 2013, 2016; Xu et al., 2016; Park C. S. et al., 2019) (30.77%, 4/13), amniotic fluid (Abdel-Maguid et al., 2019; El-Domyati et al., 2019, 2020; Prakoeswa et al., 2019) (30.77%, 4/13), umbilical cord blood (Kim et al., 2018; Kim J. et al., 2020) (15.38%, 2/13), human embryos (Seo et al., 2013; Lee S. M. et al., 2014) (15.38%, 2/13), and the placenta (Xu et al., 2016).

**TABLE 10:** Studies regarding mesenchymal stromal cell-conditioned medium for skin rejuvenation in humans.

	**MSC source**	**Method of tissue extraction**	**Type of study**	**Indication and population**	**Groups of treatments and via of administration**	**Follow-up (days)**	**Assessment**	**Main outcome**	**Other outcomes**
El-Domyati et al., 2020	Human amniotic fluid	−	Prospective observational study	Volunteers with facial aging 49.9 ± 5.59 years 3:7 (M:F)	1 ml of MSC-CM was topically applied to the treated group - Skin needling (control) - Skin needling+MSC-CM (*n* = 10)	30	Clinical examination, photographs, histology (HE, MT, Orcein stains)	The percentage of improvement was higher in the MSC-CM group compared with controls (65.40 ± 11.34 vs. 38.60 ± 9.02; *p* < 0.001)	Remodeling of the dermal structures was observed mainly on the combined side. Epidermal thickness increased on both treated sides
Kim J. et al., 2020	Human umbilical cord	Umbilical cord dissection	Randomized, investigator−blinded, prospective, split−face comparison study	Patient with large pores or wrinkles on the face that underwent laser resurfacing (42.2 years, range 25–56)	Topical application twice a day - MSC−CM cream (control) - MSC−CM cream and serum (*n* = 23)	21	Macroscopic appearance (photography, area of microcrusts), skin biophysical parameters (TEWL, SCH, erythema)	The percentage of the total microcrust area was significantly smaller in the MSC−CM cream and serum group than in the cream group (2.70 ± 0.56 vs. 3.13 ± 0.76, *p* < 0.05)	A slight increase in SCH values were observed in both groups without changes in TEWL. Patient satisfaction was similar in both groups. No adverse events were reported related to MSC-CM application
Abdel-Maguid et al., 2019	Human amniotic fluid	−	Prospective randomized split-face study	Patients with atrophic acne scars	- Group I (*n* = 17). FxCR+topical MSC-CM on one side of the face or FxCR plus saline on the other side - Group II (*n* = 16). FxCR+ topical PRP on one side of the face or FxCR+ topical MSCs on the other side	90	Macroscopic appearance (photography), histology (HE, MT), qRT-PCR	In both groups, scars improved after treatment. No significant difference in clinical improvement of acne scars was observed between the FxCR+MSC-CM and FxCR, while better and faster improvement was detected on FxCR+PRP side compared with FxCR +MSC-CM side	All patients developed transient erythema and mild edema without differences between groups. Dermal collagen was increased and procollagen type I gene was upregulated in both FCL/PRP and FCL/SC-CM sides compared with FCL only sides
El-Domyati et al., 2019	Human amniotic fluid	−	Prospective observational study	Patients with atrophic acne scars.	Topical application after five sessions of microneedling with dermaroller - 1 ml of MSCs−CM - Non-treated (*n* = 10)	90	Clinical examination, histology, histometric analysis	There was a significant increase in the improvement percentage of acne scars on the MSC−CM-treated side (65.40 ± 11.34 vs. 38.60 ± 9.02)	Improvement of character of collagen and elastic fibers was noticed, especially on MSC−CM side. Significant increase in epidermal thickness on both sides of face was detected. Erythema and slight edema appeared on both cheek sides
Park C. S. et al., 2019	Human adipose tissue	−	Prospective randomized split-face study	Patients with atrophic acne scars	Topical application twice a day after laser treatment - 80% MSC-CM+20% HA - HA (*n* = 15)	60	Scar volume and erythema were objectively evaluated using an Antera 3DVR CS	Scar volume was reduced by 23.5% in MSC-CM side vs. 15.0% in control side, and the volume of the skin pores was reduced by 37.6% in MSC-CM side vs. 15.9% in control side	The erythema increase was lower in MSC-CM side (2.8% vs. vs. 3.1%)
Prakoeswa et al., 2019	Human amniotic membrane	−	Randomized, matching pair, clinical trial	Healthy women with clinical photoageing (50.31 ± 5.1 years)	3 ml topically applied every 2 weeks after microneedling - Normal saline (control) - MSC-CM (*n* = 48)	56	Macroscopic appearance (photography, Glogau scale)	MSC-CM group showed better improvements in pore and wrinkle	Skin tone did not improve in either of the groups
Kim et al., 2018	Human umbilical cord	−	Prospective observational study	Healthy women with face wrinkles (range 18–55 years)	10% MSC-CMs in cream base topically applied daily on face skin (*n* = 22)	28	Macroscopic appearance (photography), ultrasound	Dermal density was increased by 2.46% compared before treatment	Wrinkles of eye-end area were decreased after the treatment. No irritation, stinging, or any adverse reactions were observed
Xu et al., 2016	Human adipose tissue and placenta	−	Prospective randomized clinical trial	Healthy volunteer	Intradermal injections - AT-MSC-CM+HA - P-MSC-CM+HA - HA (control) (*n* = 6/group)	15	Biophysical measurements	Erythema, melanin, elasticity, TEWL, and hydration showed improvement in hAD-MSC-CM and hP-MSC-CM compared with the control group	Only the melanin index of the hAD-MSC-CM group was significantly lower than that of the hP-MSC-CM group
Zhou et al., 2016	Human subcutaneous adipose tissue	Liposuction	Prospective observational study	Patients with facial wrinkles and patients with atrophic acne scars. 36.4 years (range 24–50) 5:6 male:female	3 ml topical application after FxCR - MSC-CM - DMEM (*n* = 9 with facial wrinkles *n* = 13 with atrophic acne scars)	90	Macroscopic appearance (photography), subjective satisfaction scale, biophysical measurements (erythema, melanin, TEWL, elasticity, skin surface roughness, hydration), and histology (HE, MT, Gomori’s aldehyde fuchsine staining)	Subjective satisfaction (2.35 ± 0.69 vs. 2.08 ± 0.76, *p* < 0.05) and objective clinical assessment (2.78 ± 0.45 vs. 1.89 ± 0.60, *p* < 0.05) were higher in MSC-CM group than DMEM	Elasticity and hydration were significantly higher in MSC-CM side, while TEWL and roughness were lower. Increased dermal collagen and elastin density were found in MSC-CM side. No adverse events were reported in the study
Lee H. J. et al., 2014	Human embryo		Prospective randomized controlled observer-blind split face study	Healthy individuals with face wrinkles (51.6 years, range 41–64)	Treatment every 2 weeks - Microneedling (control) - Microneedling + 1.5 ml of MSC-CM (*n* = 25)	84	Macroscopic appearance (photography), biophysical parameters (erythema, melanin, elasticity)	Overall satisfaction was higher in MSC-CM group than in controls (3.25 ± 1.26 vs. 2.72 ± 1.45) and also objective clinical improvement (1.92 ± 0.42 vs. 1.49 ± 0.48)	Erythema, melanin, and elasticity improved more in MSC-CM group. No serious adverse events were observed; only mild pain, erythema, and desquamation were found
Seo et al., 2013	Human embryo	−	Prospective randomized controlled, investigator-blinded, split-face study	Healthy volunteers. 53.8 ± 3.21 years, range 41–64	- Microneedling fractional radiofrequency (control) - Microneedling fractional radiofrequency+MSC-CM (*n* = 15)	28	Macroscopic appearance (photography), biophysical parameters (SCH, erythema, melanin, wrinkles, elasticity), histology (HE), IHC	More improvements of wrinkles and overall skin appearance were observed in combined treatment compared with microneedling alone (2.06 ± 0.70 for radiofrequency and 2.20 ± 0.68 for the combined treatment, *p* < 0.05)	Patients’ overall satisfaction scores were higher in combined treatment (2.35 ± 0.42 vs. 2.00 ± 0.65). SCH showed a greater increase in the combined treatment. Similar decreases in erythema and melanin index were observed in both groups. No serious adverse events were reported
Zhou et al., 2013	Human subcutaneous adipose tissue samples	Liposuction	Prospective observational study	Healthy volunteers. 24–33 years 5:14 male:female	Topical application after laser treatment: - FxCR 8 mJ, with MSC-CM - FxCR 8 mJ with DMEM - FxCR 16 mJ with MSC-CM - FxCR 8 mJ with DMEM (*n* = 19)	21	Macroscopic appearance (photography), biophysical parameters (TEWL, erythema, melanin, elasticity), histology (HE, MT, Gomori’s aldehyde fuchsine staining), qT-PCR	The MSC-CM-treated side shows less erythema and less pigmentation	MSC-CM side also showed a greater reduction of TEWL. There were no differences in elasticity parameters. The mRNA of type III procollagen in MSC-CM-treated group was 2.6 times that of control. No adverse events were reported

*AT, adipose tissue-derived; CM, conditioned medium; DMEM, Dulbecco’s modified Eagle medium; FxCR, fractional carbon dioxide laser resurfacing; HA, hyaluronic acid; HE, hematoxylin and eosin; hCB, human cord blood; hESC, human embryonic stem cell; IHC, immunohistochemistry; MSCs, mesenchymal stromal cells; MT, Masson’s trichrome; P-MSCs, placenta-derived mesenchymal stromal cells; qRT-PCR, real-time quantitative polymerase chain reaction; PRP, platelet-rich plasma.*

Two hundred six healthy volunteers were treated with MSC-CM for anti-aging therapies (Seo et al., 2013; Zhou et al., 2013; Lee S. M. et al., 2014; Xu et al., 2016; Kim et al., 2018; Prakoeswa et al., 2019; El-Domyati et al., 2020; Kim J. et al., 2020). This research included women between 24 and 64 years. The studies evaluated the effects of microneedling, radiofrequency, or laser therapy alone compared with combined treatment with MSC-CM. They observed that combined therapy showed better improvement in macroscopic appearance (less pores and wrinkles), biophysical parameters (increased SCH and decreased TEWL, erythema, and melanin), and histology (increased dermal density) and had higher patient satisfaction scores. Similar results were found for the application of CM derived from human adipose tissue and human placenta, showing only a greater decrease in melanin index in the AT-MSC-CM group (Xu et al., 2016). The application of human UC-MSC-CM containing serum and cream on patients who underwent fractional laser therapy showed less microcrusts than the application of UC-MSC-CM only containing cream (Kim J. et al., 2020). No serious adverse events were observed in these reports.

Moreover, MSC-CM was also tested on acne scars (Zhou et al., 2016; Abdel-Maguid et al., 2019; El-Domyati et al., 2019; Park C. S. et al., 2019), suggesting that combined therapy (laser+MSC-CM) may increase the regenerative effects of fractional laser. Four studies evaluated the therapeutic potential in 71 patients with a mean follow-up of 82.5 days (range 60–90). After laser treatment, topical application of MSC-CM showed greater scar volume reduction than did with the control, by 23.5% on the MSC-CM side vs. 15.0% on the control side (Park C. S. et al., 2019). Furthermore, the MSC-CM-treated side improved subjective satisfaction and clinical assessment, showing better TEWL, SCH, elasticity, roughness, and collagen density than did the controls (laser alone or DMEM). Nevertheless, laser therapy+MSC-CM showed lower clinical improvement in acne scars than the combination of laser+platelet-rich plasma (Abdel-Maguid et al., 2019). Adverse events reported were erythema and edema, linked more to laser therapy than MSC-CM treatment.

### Inflammatory Skin Diseases

#### Preclinical Studies

One study evaluated the effect of CM derived from murine adipose tissue for treating atopic dermatitis (AD) (Park H. S. et al., 2019; Table 11). Mice were followed up 33 days after AD lesions had developed and treated with subcutaneous injections every 3 days with saline, MSC, MSC-CM, or 2.5% cortisone lotion (*n* = 6/group). A higher AD severity index decrease was observed in groups treated with MSC, MSC-CM, or cortisone, as compared with the saline group. Moreover, the severity index (assessed by SCORAD) was lower in the MSC-CM group treated with MSCs or cortisone. Skin thickness decreases were similar between MSC-CM, MSC, and cortisone, and all greater than in the saline group. Likewise, mast cell infiltration was reduced in MSC-CM, MSC, and cortisone groups. Th2 expression, measured in optical densities, was lower in the MSC-CM groups than in the MSC, cortisone, or saline group (1.58 ± 1.84 vs. 3.79 ± 2.08 vs. 4.10 ± 3.32 vs. 19.19 ± 5.54). Ig E levels were slightly reduced in mice that were treated with MSC, MSC-CM, or cortisone. IL-4 was also reduced in the group treated with MSC-CM (Park H. S. et al., 2019).

**TABLE 11:** Studies regarding mesenchymal stromal cell- conditioned medium for treating inflammatory skin diseases.

	**MSC source**	**Method of tissue extraction**	**MSC characterization**	**MSC treatment**	**Model and patients**	**Groups of treatments and via of administration**	**Follow-up (days)**	**Assessment**	**Main outcome**	**Other outcomes**
**Animals**										
Park H. S. et al., 2019	Murine adipose tissue from inguinal region	Inguinal incision	Flow cytometry (CD45−, CD29+, CD105+, CD73+, CD34−, CD44+, and CD90+)	MSCs of passage 1 at 70% confluence were used. CM was then collected	Murine. AD model. 2,4-dinitrochlorobenzene was applied on the shaved dorsal skin of every mouse for 7.5 weeks to induce AD-like skin lesions	200 μl subcutaneously injected every 3 days (on days 13, 16, and 19) - Saline - MSCs - MSC-CM - 2.5% cortisone lotion (*n* = 6/group)	33	Macroscopic appearance (photography, SCORAD), histology (HE), IHC	AD severity index was significantly lower in groups treated with MSC, MSC-CM, or cortisone, compared with saline group (*p* < 0.001)	Skin thickness decreases were similar between MSC, MSC-CM, and cortisone and all greater than placebo. Mast cell infiltration was reduced in the MSC-treated group (40.20 ± 9.44 cells, *p* < 0.001), MSC−CM (47.33 ± 13.13 cells, *p* < 0.001), or cortisone (51.00 ± 21.46 cells, *p* < 0.001) compared with saline (134.00 ± 7.42 cells). Thymic stromal lymphopoietin expression level in saline group was higher (11.7 ± 1.69) than in MSC group (5.07 ± 0.76, *p* < 0.001), MSC−CM (4.52 ± 1.33, *p* < 0.001), or cortisone group (6.72 ± 1.66, *p* < 0.001). CD45 expression was elevated in saline−treated group (1.68 ± 0.29), but it was reduced in MSC group (0.38 ± 0.18, *p* < 0.001), MSC−CM (0.19 ± 0.17, *p* < 0.001), or cortisone (0.37 ± 0.15, *p* < 0.001). Moreover, TH2 expression levels were lower in MSC-treated mice (3.79 ± 2.08, *p* < 0.001), MSC−CM (1.58 ± 1.84, *p* < 0.001), or cortisone (4.10 ± 3.32, *p* < 0.001) compared with saline group (19.19 ± 5.54). CXCL9 expression levels were lower in MSC group
										(4.49 ± 1.25, *p* < 0.01), MSC−CM (8.78 ± 0.51, *p* < 0.01), or cortisone (15.83 ± 3.94, *p* < 0.05) compared with saline group (41.2 ± 4.15). CCL20 expression was higher in saline−treated group (61.18 ± 1.97), but it was reduced by MSCs (23.99 ± 2.46, *p* < 0.001), MSC−CM (25.98 ± 3.19, *p* < 0.001), or cortisone (28.87 ± 3.1, *p* < 0.001). IgE levels were slightly reduced in mice that were treated with MSC, MSC-CM, or cortisone. Tissue levels of IFN−γ were reduced MSCs (66.20 ± 14.51 pg/ml, *p* < 0.001) or MSC−CM (36.27 ± 3.26 pg/ml, *p* < 0.001) compared with saline (117.14 ± 45.84 pg/ml). Similarly, serum levels of IL−33 were lower in MSC group (32.20 ± 3.04 pg/ml; *p* < 0.001) and MSC−CM group (42.83 ± 6.07 pg/ml, *p* < 0.001) than in saline group (64.06 ± 8.58 pg/ml). IL-4 level in the MSC-CM group (30.03 ± 8.39 pg/ml, *p* < 0.001) was decreased compared with saline (51.97 ± 2.24 pg/ml). Serum IL−13 level was decreased in MSCs−treated group (0.27 ± 0.03 pg/ml,
**Human**										
Kim Y. J. et al., 2020	Human umbilical cord	Umbilical cord dissection	Flow cytometry (CD24+, CD29+, CD44+, CD73+, CD90+, CD105+, CD10−, CD14−, CD31−, CD34−, CD45−, CD62−, CD133−, HLA-DR−). Osteogenic, adipogenic, and chondrogenic differentiation	MSCs of passage 6 were used. CM was collected	Prospective observational study Patients with atopic dermatitis patients. Age 24.68 ± 4.32 Sex 15:13 male:female	MSC-CM containing cream bases were applied twice a day to patients’ lesion and non-lesion skin (*n* = 28)	28	Skin biophysical parameters (TEWL, SCH)	MSC-CM improved skin barrier functions	*p* < 0.05) compared with saline group (3.68 ± 1.40 pg/ml) SCH increased and TEWL decreased on both the lesion and non-involved skin after MSC-CM. SCH increased by 15.67 and 14.49 AU on the lesion and non-involved skin, respectively. TEWL decreased 15.09 AU and 3.08 AU on the lesion and non-involved skin, respectively
Seetharaman et al., 2019	Human adipose tissue from the waist area	Lipoaspiration	−	MSCs of passage at 90% confluence were used. CM was then collected	Case report. Patient with scalp psoriasis. 38 years Male	MSC-CM was topically applied on the lesions once a day	30	Clinical examination (PSSI)	Psoriatic plaques were reduced after MSC-CM treatment	PSSI score reduced from 28 to 0 and diseases regression continued for 6 months of follow-up

*AD, atopic dermatitis; CM, conditioned medium; HE, hematoxylin and eosin; IHC, immunohistochemistry; MSCs, mesenchymal stromal cell; PSSI, Psoriasis Scalp Severity Index; SCORAD, SCORing Atopic Dermatitis Index; SCH, stratum corneum hydration, TEWL, transepidermal water loss.*

#### Clinical Studies

One study evaluated the effects of MSC-CM for treating AD (Kim Y. J. et al., 2020), and one case report showed the outcomes in a patient with scalp psoriasis treated with MSC-CM (Seetharaman et al., 2019; Table 11).

Twenty-eight patients (15 males and 13 females; mean age 24.68 ± 4.32 years) were treated with human UC-MSC-CM in cream bases for 4 weeks. MSC-CM was applied on both eczematous lesions and non-involved skin twice a day. After treatment, SCH increased by 15.67 arbitrary units (AU), a relative unit of measurement to show the ratio of amount of some quantities to a predetermined reference measurement, on eczematous lesions and 14.49 AU on non-involved skin. Moreover, TEWL decreased by 15.09 g⋅m^−2^⋅h^−1^ AU on eczematous lesions and 3.08 g⋅m^−2^⋅h^−1^ on non-involved skin (Kim Y. J. et al., 2020). In agreement with preclinical studies, these data showed that cosmetics that contained MSC-CM improved the skin barrier function and could be an effective treatment for AD patients.

One study also evaluated the clinical efficacy of MSC-CM derived from human adipose tissue for treating psoriasis scalp. A 38-year-old male was treated topically with MSC-CM on the lesions once a day. After 1-month follow-up, the number of psoriatic plaques was reduced and Psoriasis Scalp Severity Index (PSSI) changed from 28 to 0. Moreover, no relapse or adverse events were observed after 6 months’ follow-up (Seetharaman et al., 2019).

## Discussion

**FIGURE 2:**
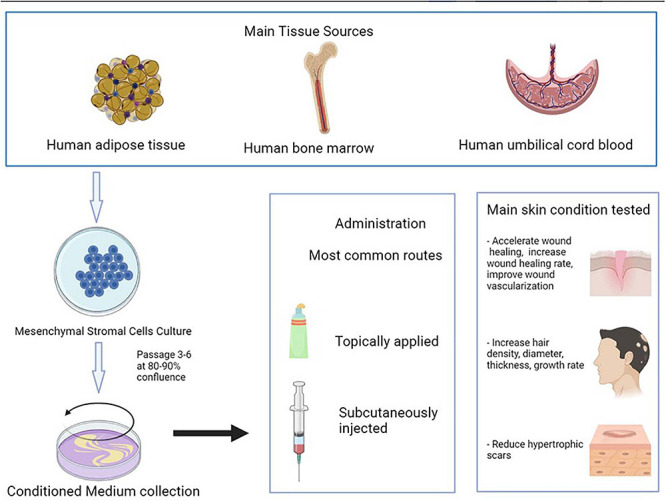
Iconographic summary of this review’s main findings.

MSC-CM may be an alternative, safe, and easily delivered therapy for several skin conditions, but evidence in clinical studies is limited. They have been mainly tested in hair restoration (Shin et al., 2015; Lee et al., 2020; Narita et al., 2020; Oh et al., 2020) and skin rejuvenation (Abdel-Maguid et al., 2019; El-Domyati et al., 2019; Kim J. et al., 2020), with promising results. Preclinical studies are mainly focused on wound healing (Shin et al., 2015; Lee et al., 2020; Narita et al., 2020; Oh et al., 2020), showing high rates in wound closure. MSC-CM may also be an effective treatment for decreasing hypertrophic scars (Arjunan et al., 2020; Hu et al., 2020), improving flap reperfusion (Mirabella et al., 2012; Lee S. M. et al., 2014; Cooper et al., 2018; Pu et al., 2019), and treating psoriasis (Seetharaman et al., 2019) and AD (Park H. S. et al., 2019; Kim Y. J. et al., 2020). Nevertheless, there was significant variability between studies in the cell source, cell treatment, method of delivery, disease model used to assess efficacy, ways of assessment, and outcome evaluation. This review provides a useful summary regarding the use of MSC-CM for skin conditions and highlights the need to standardize manufacturing processes and characterize CM. The main findings of this review are summarized in Figure 2.

It is important to characterize the composition of CM, but there is a lack of evidence in this aspect. Some studies showed that CM contained high concentrations of growth factors and cytokines associated with angiogenesis and endothelial cell migration such as EGF, tissue inhibitors of metalloproteinase-1, or IGF-binding protein-7 (De Gregorio et al., 2020; Kim Y. J. et al., 2020). The properties of MSC-CM vary depending on the cell source (Sagaradze et al., 2019). More than 10 sources of MSCs were used to treat skin conditions. MSCs were mainly isolated from human adipose tissue, human bone marrow, and human umbilical cord blood. Another important aspect is the timing of the collection of CM from the cells and concentration steps carried out for obtaining CM (Im et al., 2019). Most studies used cells from passages 3 to 6 at 80–90% confluence. The secretome of cells may also vary depending on the age of the cells (Maguire, 2013). Furthermore, the same type of cells is reported to secrete different levels of paracrine factors depending on the culture condition (Park et al., 2010) (hypoxic vs. normoxic) and the scaffold type (Li T. et al., 2019) (monolayer vs. 3D cultures). Hypoxic treatment is one of the most frequently used ways to improve CM, as hypoxic stress reduces oxygen and improves cellular functions (Du et al., 2017). CM collected from MSCs cultured under hypoxic conditions improved the cells’ capacities of proliferation and self-renewal (Chen et al., 2014; Jun et al., 2014; Du et al., 2016a, 2017). Determining the levels of paracrine factors secreted by MSCs at different passages with different conditions may also provide knowledge about the best stromal cell growth stage for obtaining a specific group of paracrine factors. Furthermore, MSC-CM should be considered both an alternative therapy and a synergic therapy for improving skin conditions, as MSC-CM combined with the conventional therapy has been proved to have better results than MSC-CM or the conventional therapy alone (Pouriran et al., 2016; Amini et al., 2018; Bagheri et al., 2018; Fridoni et al., 2019; Kouhkheil et al., 2019). In fact, some studies regarding wound healing in diabetic and infected wounds showed that PBM+MSC-CM application accelerated the process of wound healing, while it did not happen when using MSC-CM alone (Pouriran et al., 2016; Fridoni et al., 2019; Kouhkheil et al., 2019).

Several routes of administration have been also tested. Topical application and subcutaneous injection of CM were the most common routes of administration, and they are also the least invasive ways, appearing as promising routes for future research on MSC-CM.

No adverse events were reported to be related to MSC-CM in either preclinical or clinical trials. This is one of the main advantages of using MSC-CM instead of CM, as adverse events related to cells such as hyperimmunogenicity or tumorigenicity can be avoided (Gunawardena et al., 2019b). Only three studies evaluated differences between MSCs and MSC-CM, showing similar results between them (Tamari et al., 2013; Park H. S. et al., 2019; Zhang S. et al., 2020). Similar effectiveness was observed between UC-MSCs and UC-MSC-CM for accelerating wound healing rate. The wound area of UC-MSC and UC-MSC-CM was ≈15% reduced as compared with controls without differences in UC-MSC and UC-MSC-CM (Park H. S. et al., 2019; Zhang S. et al., 2020). Other research also showed similar epithelialization rate when using MSC or MSC-CM (Tamari et al., 2013). Moreover, it was observed that the effect of autologous AT-MSCs and AT-MSC-CM was similar for treating AD in mouse and higher than in the control group (Park H. S. et al., 2019).

It is also important to differentiate MSC-CM from culture media only. MSC-CM is the one in which MSCs have been grown for a period of time and with some requirements, and the culture media only is the media in which we grow the MSC-CM but has not been exposed to the cells. The culture media were used as control in some studies to prove the effectiveness of MSC-CM in wound healing (Jun et al., 2014; Deng et al., 2019; Rong et al., 2019). Higher wound healing rates and faster healing were observed with MSC-CM treatment than DMEM. Moreover, it was observed that hypertrophic scar reduction was higher using MSC-CM than DMEM (Zhang et al., 2015; Liu et al., 2018; Hu et al., 2020). It was also found that the topical application of MSC-CM after laser treatment showed less erythema and less pigmentation than the application of DMEM (Zhou et al., 2013, 2016). This might mean that the effect observed of MSC-CM in these diseases is due to the secretome and is not being caused by the culture media alone.

One of the limitations of MSC-CM is that, to translate its use to patients, it is necessary to know the exact composition of each CM and that its use validation should be conducted for every disease it is to be used for. Moreover, CM needs to be given more frequently than MSC, as the half-lives of cytokines and growth factors are mostly shorter than those of stromal cells, which may survive for rather long periods (Pawitan, 2014). Further research is needed to determine the components of MSC-CM that improves skin diseases. Moreover, regulatory requirements for manufacturing, standardization, and quality control of MSC-CM regarding MSC origin, isolation methods, and culture conditions are necessary to establish the safety and efficacy profiles of these products (Park H. S. et al., 2019).

Although there are some reviews regarding MSC-CM (Pawitan, 2014; Mizukami and Yagihashi, 2016; Bogatcheva and Coleman, 2019), they include little information about cutaneous disease. Pawitan et al. included some studies where MSC-CM was used for skin repair and alopecia. Bogatcheva and Coleman only included two articles where MSC-CM was used for skin diseases, one on treating dermatitis and the other on burns. Mizukami and Yagihashi reviewed the status of preclinical studies on stromal cell therapy for diabetic polyneuropathy, but they focused on MSCs and did not include information on other diseases. As far as we know, here, we review the use of MSC-CM in all skin conditions reported in the literature for the first time.

In conclusion, MSC-CM is a promising therapy for skin conditions. Studies on animals showed important rates of wound closure after MSC-CM treatment and clinical studies showed good results for skin rejuvenation. Further studies are needed to corroborate safety and effectiveness and to standardize CM manufacturing.

## Author Contributions

TM-V and SA-S contributed to conceptualization, formal analysis, investigation, and writing – original draft preparation. TM-V, ÁS-S, and SA-S contributed to methodology. AM-L contributed to software. TM-V, AM-L, and SA-S contributed to validation and visualization. MQ-V contributed to resources. TM-V, ÁS-S, MS-D, and MQ-V contributed to data curation. TM-V, SA-S, ÁS-S, and AM-L contributed to writing – review and editing. All authors have read and agreed to the published version of the manuscript.

## Conflict of Interest

The authors declare that the research was conducted in the absence of any commercial or financial relationships that could be construed as a potential conflict of interest.

## Publisher’s Note

All claims expressed in this article are solely those of the authors and do not necessarily represent those of their affiliated organizations, or those of the publisher, the editors and the reviewers. Any product that may be evaluated in this article, or claim that may be made by its manufacturer, is not guaranteed or endorsed by the publisher.

## Abbreviations

AD: atopic dermatitis
AGA: androgenetic alopecia
AT: adipose tissue
BM: bone marrow
Botox: botulinum toxin type A
CM: conditioned medium
DMEM: Dulbecco’s modified Eagle medium
ECM: extracellular matrix
EV: extracellular vesicles
Exos: exosomes
HB-EGF: heparin binding-epidermal growth factor-like growth factor
HDFs: human dermal fibroblasts
HGF: hepatocyte growth factor
IGF: insulin-like growth factor
IL: interleukin
Men: menstrual blood
MSCs: mesenchymal stromal cells
PBS: phosphate-buffered saline
PSSI: Psoriasis Scalp Severity Index
qRT-PCR: real-time quantitative polymerase chain reaction
SCH: stratum corneum hydration (SCH)
SCORAD: SCORing Atopic Dermatitis Index
SHSs: *Haliclona* sp. spicules
SDF − 1: stromal cell-derived factor 1
TEWL: transepidermal water loss
TGF- β: transforming growth factor β
Th2: lymphocyte T helper type 2
TNF- α: tumor necrosis factor α
UC: umbilical cord
VEGF: vascular endothelial growth factor.
