# Supplementary material for: Mesenchymal Stromal Cell-Conditioned Medium for Skin Diseases: A Systematic Review
Source: Front Cell Dev Biol. 2021 Jul 23;9:654210. doi: 10.3389/fcell.2021.654210 (PMC8343397; doi:10.3389/fcell.2021.654210)
Supplement: Supplementary file 2 [file Table_1.pdf]

Table S1. Studies regarding mesenchymal stem cell- conditioned medium for treating wounds combined with other treatmetns.

	MSC source	Method of tissue extraction	MSC characterisation	Preparation of MSC-CM	Model	Groups of treatments and via of administration	Follow-up (days)	Assessment	Main outcome	Other outcomes
**Bagheri M et al. 2018**	Human bone marrow	Aspiration	Flow cytometry (CD73+, CD90+, CD105+, CD34-, CD45-)	MSCs of passage 4 at 80% confluence were used. CM was collected.	Murine. Diabetic rat model. Full-thickness skin wounds, 12 mm, on upper back.	PBM was administered once daily, 6 days per week. CM was administrated day 0 and 1 intraperitoneally.<br>- DMEM vehicle (control),<br>- MSCs-CM,<br>- PBM<br>- PMB+ MSCs-CM.<br>(n=18/group)	15	Stereological methods, tensiometric examination	MSCs-CM and PBM+ MSCs-CM increased the tensiometric properties compared to DMEM and PBM.	MSCs-CM, PBM and PBM+ MSCs-CM groups showed a significant decrease in the three types of mast cells and in the total number of mast cells compared with controls
**Amini A et al. 2018**	Human bone marrow	Aspiration	Flow cytometry (CD73+, CD90+, CD105+, CD34-, CD45-)	MSCs of passage 4 at 80% confluence were used. CM was collected.	Murine. Diabetic rat model. Full-thickness skin wounds, 12 mm, on upper thoracic and lumbar regions.	PBM was administered once daily, 6 days per week. CM was administrated day 0 and 1 intraperitoneally.<br>- DMEM vehicle (control),<br>- MSCs-CM,<br>- PBM<br>- PMB+ MSCs-CM.<br>(n=18/group)	15	Stereological methods, tensiometric examination, qRT-PCR	All treated groups significantly enhanced wound healing compared to controls. The extent of healing was significantly greater in the MSCs-CM+PBM group.	Number of fibroblast and epidermal cells, the lengths of blood vessels, bFGF and SDF-1α expression were significantly higher in MSCs-CM+PBM group.
**Pouriran R et al. 2016.**	Human bone marrow	Aspiration	Flow cytometry (CD105+, CD90+, CD73+, CD34-, CD45-).	MSCs of passage 4 at 80% confluence were used. CM was then collected.	Murine. Diabetic rat model. Full-thickness skin wounds, 12mm, on the thoracic and lumbar regions.	PWLLLT was administered once daily, 6 days per week. MSCs-CM was administrated twice intraperitoneally<br>- Non treated,<br>- MSCs-CM,<br>- PWLLLT,<br>- MSCs-CM+PWLLLT<br>(n=7/group)	15	Macroscopic appearance (photography), biomechanical examination.	PWLLLT and MSCs-CM, alone or in combination, improved biomechanical parameters in the wound.	PWLLLT was more effective compared to MSCs-CM.
**Kouhkheil R et al. 2019**	Human bone marrow	Aspiration	Flow cytometry	MSCs of passage 4 at 80% confluence were used. CM was collected.	Murine. MRSA rats infected. Full-thickness excisional wound, 15 mm, on the back.	PBM was administeredonce daily, 6 days per week. 50 μl of the 10-fold CM were administrated from day zero until day 3.	15	Clinical observation, microbiological, tensiometrical, and	There was a significant decrease in colony-forming units in PBM+ MSCs-CM and PBM groups compared to controls.	PBM+MSCs-CM, PBM, and MSCs-CM groups significantly increased wound strength compared with the control group. The PBM+ MSCs-CM and PBM groups had more stable MCs, less significant degranulated and

						-Control<br>- PBM<br>- MSCs-CM<br>- PBM + MSCs-CM<br>(n=18/group)		stereological analyses		disintegrated MCs and less significant total number of MCs compared with the control group.
**Fridoni M et al. 2019**	Human bone marrow	Aspiration	Flow cytometry (CD105+, CD90+, CD73+, CD34-, CD45-).	MSCs of passage 4 were used. CM was collected.	Murine. MRSA diabetic rats infected. Full‐thickness wound, 15mm diameter round, on the back.	PBM was administered once daily, 6 days per week. 500 µl of the 10-fold CM were injected intraperitoneally daily from day 0 until day 3.<br>-Control group,<br>- PBM,<br>- MSCs-CM<br>- PBM+ MSCs-CM.<br>(n=18/group)	15	Histology (HE), IHC	PBM+ MSCs-CM hastened wound healing process.	PBM+ MSCs-CM, MSCs-CM, and PBM groups showed a decrease in the number of neutrophils and macrophages and an increase in the number of fibroblasts and angiogenesis compared with those of the control group.

AT, Adipose tissue-derived; bFGF, basic fibroblast growth factor; AF, Amniotic fluid; BM, bone marrow; CM, Conditioned Medium; DFX, deferoxamine; DMEM, Dulbecco's Modified Eagle Medium; DP, dental pulp; EC: Endothelial-differentiated; ECM, extracellular matrix; EGF, epidermal growth factor; ELISA, Enzyme-linked immunosorbent assay; EVs, extracellular vesicles; exos; exosomes; HE, haematoxylin and eosin; hCB: human cord blood; hESC, human embryonic stem cell; IHC, immunohistochemistry; IF, immunofluorescence; MPF, micro-nano polylactic acid electrospun fibre; MSCs, Mesenchymal Stem Cells; MT, Masson's Trichrome; PBM, photobiomodulation; PBS, phosphate-buffered saline; PWLLLT, Pulsed Wave Low-Level Laser Therapy; qRT-PCR, real-time quantitative polymerase chain reaction; SVF, stromal vascular fraction; UCM, unconditioned medium; WB, Western blot; WJ, Wharton's jelly.
